# Emergence of *Nido*-Icosahedral Superatomic Units in Iridium-Doped Silver Cluster Assemblies

**DOI:** 10.1021/acs.inorgchem.6c03151

**Published:** 2026-08-17

**Authors:** Tzu-Hao Chiu, Michael N. Pillay, Jose V. Rival, Yoshiki Niihori, Yuichi Negishi, Samia Kahlal, Jean-Yves Saillard, C. W. Liu

**Affiliations:** † Department of Chemistry, 63373 National Dong Hwa University, No. 1, Sec. 2, Da Hsueh Rd. Hualien 97401, Taiwan; ‡ 13101 Institute of Multidisciplinary Research for Advanced Materials Tohoku University, Katahira 2-1-1, Aoba-ku, Sendai 980-8577, Japan; § Faculty of Textile Science and Technology, Shinshu University Tokida, Ueda, Nagano 386-8567, Japan; ∥ Univ Rennes CNRS, ISCR-UMR 6226, Rennes 35000, France

## Abstract

The concept of superatomic building blocks provides a powerful framework for understanding the stability of nonspherical metal nanoclusters; however, assemblies derived from unconventional motifs remain largely underexplored. Herein, we report a series of iridium-doped, silver-rich 16-electron superclusters (nanoclusters constructed from the assembly of individual superatomic building units), (IrH_2_)_2_Ag_31_[S_2_P­(OPr)_2_]_17_ (**1**), (IrH)­(IrH_2_)­Ag_32_[S_2_P­(OPr)_2_]_17_ (**2**), and (IrH)_2_Ag_33_[S_2_P­(OPr)_2_]_17_ (**3**), whose cores can be described as assemblies of two 8-electron superatomic units. Single-crystal X-ray diffraction, complemented by density functional theory (DFT) calculations, reveals that clusters **1** and **2** feature an unprecedented eight-electron nido-icosahedral core, IrH_2_Ag_11_, which arises as a defective motif induced by multiple hydrides. The three isolated clusters establish a formal structural relationship in which each successive composition differs by one Ag atom and one hydride, highlighting the relationship among hydride incorporation, electron count, and structural topology in superclusters. Furthermore, these clusters exhibit systematic variations in their spectroscopic properties, including a progressive red shift of their lowest-energy absorption bands, consistent with their tunable electronic structures. This work not only identifies a new type of superatomic building unit but also demonstrates that hydrides can act as key regulators of both electronic configuration and structural topology, providing important insights into how hydride-induced defects can be harnessed to expand the structural diversity and electronic modularity of noble metal nanoclusters.

## Graphical abstract

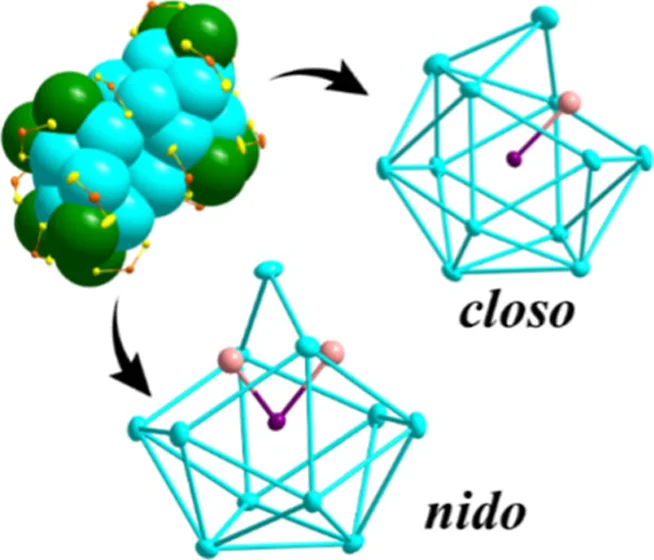

## Introduction

The superatom concept has long been employed to rationalize the stability of ligand-protected metal nanoclusters.^1–5^ It is derived from the spherical jellium approximation,^6^ which takes advantage of the spheroidal and compact nature of the nanocluster core, allowing the latter to be described as a kind of giant pseudoatom. It follows that the electronic structure associated with the metal (*n* + 1)­s valence electrons of such a superatom somewhat mimics that of an atom, with superatomic shells ordered as 1S < 1P < 1D < 2S < 1F < 2P < 1G... As with an atom, chemical stability of a superatom is generally ensured when complete filling of superatomic levels occurs, that is, for the so-called magic electron counts of 2, 8, 18, 20, 34... Over the years, numerous 8-electron nanoclusters (1S^2^ 1P^6^ configuration) of group 11 metals have been reported,^7–10^ almost all of them containing a spheroidal and close-packed M@M_12_ centered icosahedral core. In recent years, doping the superatomic core with noble metals (M′) has become one of the most actively explored research avenues. Such doping not only modulates the various properties of the clusters, enabling the development of more diverse functional materials, but can also enhance their structural stability, thereby increasing their potential for practical applications.^10–18^ In this context, (M′H_*x*_)@M_12_ species containing *x* = 1, 2, or even 3 encapsulated hydride(s), whose electron(s) are part of the 8-electron count, have also been reported.^16–23^

Nanoclusters with nonspheroidal cores also exist. By far the largest family of these systems is constructed from the assembly of centered icosahedral superatomic building blocks through vertex-, edge-, or face-sharing modes. The resulting supercluster frameworks^24–29^ are associated with electron counts which can be rationalized in terms of closed-shell assemblies of superatomic building blocks that follow the octet (possibly 18-electron^30^) rule.^31–37^ Most of them are actually supercluster equivalents of van der Waals pairs of atoms (isolobal to closed-shell dimers like Ne_2_), as exemplified by the many Ag- and Au-based cluster of clusters systems made of two 8-electron superatomic units.^37–48^

It is of note that nanoclusters having one (or more) vacancy on their superatomic core can also exist, despite their nonperfectly spheroidal shape.^47,49–52^ Some time ago, we reported clusters [MHCu_11_{S_2_P­(O^*i*^Pr)_2_}_6_(CCPh)_4_] (M = Pd, Pt),^53,54^ whose metallic core is an MH-centered cuboctahedron which is missing a vertex. These defective (MH)@(Cu_11_□) (□ = vacancy) species are 2-electron superatoms, with an electronic structure resembling that of their nondefective spheroidal 2-electron Cu@Cu_12_ parents. The relationship with the boranes and organometallic Wade–Mingos clusters^55,56^ is obvious: removing one vertex from a nondefective spheroidal cluster (*closo* species) to generate a defective *nido* species does not change its favored closed-shell electron count. Herein, we report the first superatomic assembly constructed from icosahedral cores having one missing vertex (*nido*-type), namely, three isoelectronic iridium-doped silver-rich superclusters of general formula Ir_2_H_*x*_Ag_35–*x*_[dtp]_17_ (*x* = 4, 3, 2; dtp = dithiophosphate): (IrH_2_)_2_Ag_31_[S_2_P­(OPr)_2_]_17_ (**1**), (IrH)­(IrH_2_)­Ag_32_[S_2_P­(OPr)_2_]_17_ (**2**), and (IrH)_2_Ag_33_[S_2_P­(OPr)_2_]_17_ (**3**), fully characterized by single-crystal X-ray diffraction and complementary analytical techniques. All three clusters share an identical passivating peripheral shell, [Ag_10_(dtp)_17_]^7–^, but possess distinct 16-electron cores. Notably, the building units in **1** and **2** contain an extremely rare 8-electron *nido*-icosahedral core. This discovery further enriches the superclusters’ structural diversity.

## Results and Discussion

Compounds **1**–**3** were synthesized through a one-pot reaction in THF using an Ir/Ag/L/H ratio of 1:15:8:15, followed by purification via column chromatography and TLC (Figure S1). The isolated products were readily distinguished by ESI-MS owing to their different metal nuclearities. For **1**, the most intense peak corresponds to [**1** + Ag]^+^ (exp: 7465.1924 Da; calcd: 7465.2022 Da). In the spectrum of **2**, the dominant peak is shifted relative to **1**, consistent with the addition of one Ag atom accompanied by the loss of one hydride. A similar trend is observed for **3**, whose main signal indicates one additional Ag atom and one fewer hydride compared to **2** (Figure 1a–c). To confirm the presence of hydrides, the deuterated analogues of **1**_**D**_, **2**_**D**_, and **3**_**D**_ were synthesized using [BD_4_]^−^ in place of [BH_4_]^−^. Their ESI-MS spectra exhibit clear mass shifts relative to those of the corresponding hydride-containing clusters. In each case, the dominant signals correspond to the [M + Ag]^+^ ions. Compared with their hydride analogues, the peaks for **1**_**D**_, **2**_**D**_, and **3**_**D**_ are shifted by approximately **3**, **2**, and **1** Da, respectively, indicating successful incorporation of deuterium into the clusters. The observed mass shifts suggest that the detected ions predominantly contain one fewer deuterium atom than the fully deuterated compositions, possibly due to partial deuteration of the reducing agent NaBD_4_ (D, 98%) or H/D exchange during sample handling or ionization (Figure S2). The three clusters display similar absorption profiles, with the lowest-energy band located in the 600–700 nm region. This observation is consistent with previously reported dtp-protected 16-electron clusters.^44,45^ Notably, the lowest-energy band progressively red-shifts with increasing Ag nuclearity, appearing at 609, 630, and 652 nm for **1**–**3**, respectively (Figure 1d).

**Figure 1:**
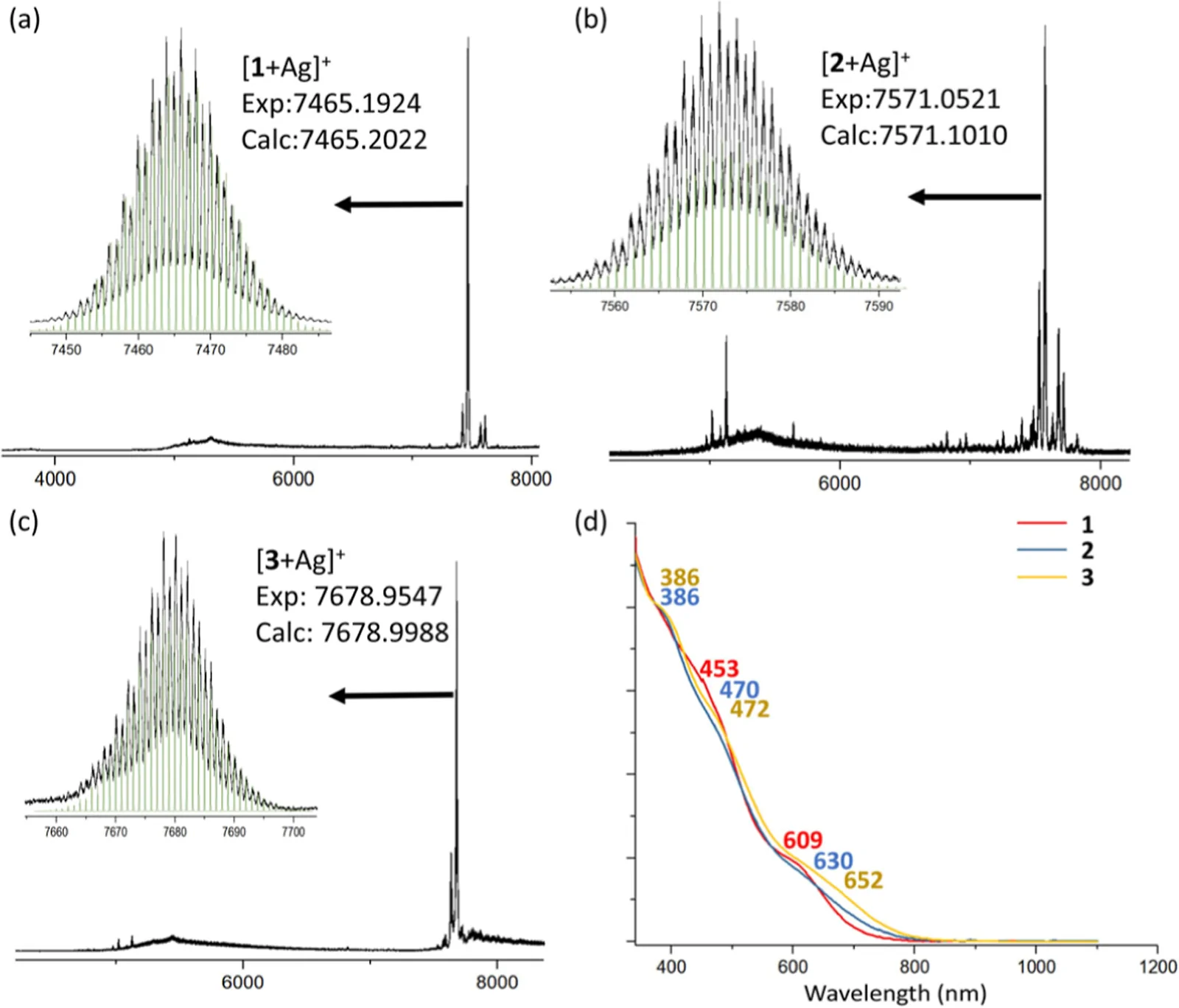
Positive-mode ESI mass spectrum of (a) **1**, (b) **2**, and (c) **3**. Insets: experimental (black) and simulated (green) isotopic distribution of the ion peak [M + Ag]^+^. (d) Absorption spectra of **1**, **2**, and **3** (2-MeTHF, RT).

The molecular structures of compounds **1**–**3** (Figure 2) were determined by single-crystal X-ray diffraction. Selected metrical data are given in Table 1. Crystal data and structure refinement details are provided in Table S1. All three clusters feature a bis-icosahedral Ir_2_H_*x*_Ag_25–*x*_ core protected by an outer shell composed of 10 capping silver atoms and 17 dtp ligands. The hydride positions in the three clusters were first determined by density functional theory (DFT) calculations and subsequently incorporated into the crystallographic model as fixed hydrogen atoms during the X-ray refinement. Because of the dominant scattering from the surrounding Ag and Ir atoms, the hydride positions could not be reliably identified from the difference Fourier maps alone. They were found fully consistent with the distorted shapes of their icosahedral cages. The core of **1** consists of two IrH_2_Ag_11_ units sharing a single vertex, forming a *C*_2_ pseudosymmetric (IrH_2_)_2_Ag_21_ framework, with the *C*_2_ axis perpendicular to the Ir–Ir vector (Figure 2a,e). Notably, the IrH_2_Ag_11_ units represent a rare *nido*-type icosahedral framework (one missing vertex, (IrH_2_)@(Ag_11_□) which, to the best of our knowledge, has not been previously reported. In **2**, the core evolves to (IrH_2_)­(IrH)­Ag_22_, which can be described as a vertex-sharing assembly of one *nido*-type (IrH_2_)@(Ag_11_□) and one *closo*-type (complete) (IrH)@(Ag_12_) icosahedral units, leading to a *C*_1_ asymmetric arrangement (Figure 2b,e,f). The core of **3** is composed of two vertex-sharing *closo*-type (IrH)@(Ag_12_) units, forming a *C*_2_ pseudosymmetric structure. This motif closely resembles the 16-electron core reported in (RhH)_2_Ag_33_(dtp)_17_ ^45^ (Figure 2c,f) in the three clusters; the [Ag_10_(dtp)_17_]^7–^ shell retains an overall *C*_2_ pseudo-symmetry, reminiscent of those previously reported systems such as (RhH)_2_Ag_33_(dtp)_17_ and Pt_2_Ag_33_(dtp)_17_ ^44,45^ (Figure 2d); while the *closo*-type (IrH)@(Ag_12_) unit has previously been reported in IrHAg_24_ and IrHAg_20_,^20,21^ the *nido*-type (IrH_2_)@(Ag_11_) unit reported herein is unprecedented. The ten Ag caps are evenly distributed over the triangular faces at both ends of the rod-shaped core, each adopting a typical Ag­(I) trigonal planar coordination geometry with three sulfur atoms from the ligands. Two ligands cap the terminal positions of the rod-shaped core, while the remaining 15 are evenly divided into three sets, each forming a belt-like ring that wraps around the core perpendicular to the long axis (Figure S3); to gain further insight into the origin of the *nido*-icosahedral core, a detailed structural analysis was carried out. It is observed that, in all 16-electron bi-icosahedral systems, except (RhH)_2_Ag_33_(dtp)_17_, the Ag atoms belonging to the icosahedral unit predominantly coordinate to only one nonmetal atom, typically a ligand sulfur or halogen atom (Figure S4).^36,41–48^ However, in **2** and **3**, a rare coordination mode is observed, in which a specific Ag atom of each distorted *closo*-icosahedral unit (labeled Ag_ico(S2)_ in the following) is bound to two sulfur atoms and one hydride, resembling the coordination environment typically found for Ag­(I) species (Figure S5). Because the Ag atoms of the 16-electron core tend to adopt low coordination numbers toward nonmetal ligands, Ag_ico(S2)_ becomes more susceptible to removal. The three isolated clusters establish a formal structural relationship in which each successive composition differs by one Ag atom and one hydride, highlighting the relationship between hydride incorporation, electron count, and structural topology in superatomic assemblies. We therefore propose that incorporation of an additional hydride into an eight-electron superatomic unit may promote redistribution of the metal atoms within the cluster. In one possible pathway, Ag_ico(S2)_ is directly removed from the icosahedral framework, likely as an Ag^+^ species. Alternatively, the process may proceed via initial removal of an Ag_cap_, followed by migration of Ag_ico(S2)_ from the core to occupy the vacant capping position (Figures S6 and S7). Both pathways ultimately allow the cluster to accommodate the additional hydride while maintaining overall charge neutrality, leading to the formation of the observed *nido*-IrH_2_Ag_11_ core.

**Figure 2:**
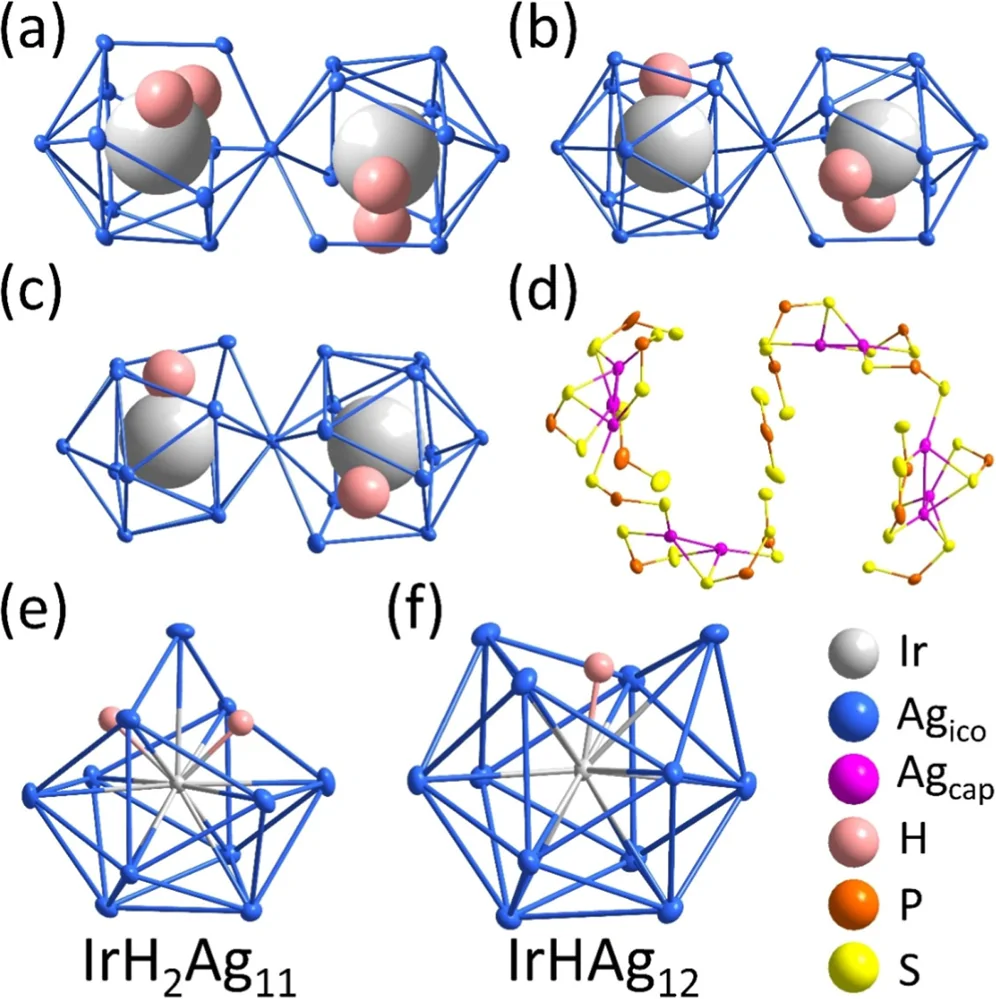
(a) (IrH_2_)_2_Ag_21_ core of **1**, (b) (IrH)­(IrH_2_)­Ag_22_ core of **2**, (c) (IrH)_2_Ag_23_ core of **3**, (d) [Ag_10_(dtp)_17_]^7–^ shell, (e) IrH_2_Ag_11_ nido-icosahedral core, and (f) IrHAg_12_ icosahedral core.

**Table 1:** Selected Experimental and Computed Distances (Å), Wiberg Bond Indices (WBIs, in Brackets), and Angles (deg) for **1**–**3**

	(IrH_2_)_2_Ag_31_[S_2_P(OPr)_2_]_17_ (**1**)	(IrH_2_)(IrH)Ag_32_[S_2_P(OPr)_2_]_17_ (**2**)	(IrH)_2_Ag_33_[S_2_P(OPr)_2_]_17_ (**3**)
SCXRD			
Ir–Ag_ico_	2.712(1)–2.878(2)	2.727(1)–3.160(3)	2.709(1)–3.155(1)
	avg. 2.787(1)	avg. 2.804(2)	avg. 2.805(1)
Ag_ico_–Ag_ico_	2.768(2)–3.016(2)	2.712(2)–3.140(10)	2.708(1)–2.999(2)
	avg. 2.892(2)	avg. 2.910(3)	avg. 2.899(1)
Ag_ico_–Ag_cap_	2.876(2)–3.183(2)	2.869(2)–3.166(2)	2.830(2)–3.170(2)
	avg. 2.989(2)	avg. 2.987(2)	avg. 2.985(2)
DFT			
Ir–Ag_ico_	2.818–2.981 [0.059–0.199]	2.808–3.130 [0.062–0.217]	2.805–3.154 [0.080–0.212]
	2.879 [0.150]	2.884 [0.160]	2.886 [0.168]
Ag_ico_–Ag_ico_	2.866–3.074 [0.028–0.127]	2.866–3.131 [0.026–0.124]	2.893–3.177 [0.023–0.125]
	2.988 [0.070]	2.989 [0.070]	2.992 [0.069]
Ag_ico_–Ag_cap_	2.969–3.321 [0.025–0.056]	2.948–3.297 [0.026–0.058]	2.953–3.310 [0.027–0.058]
	3.094 [0.045]	3.091 [0.044]	3.087 [0.045]
Ir–H	1.659–1.667 [0.328–0.346]	1.647–1.678 [0.305–0.371]	1.671–1.672 [0.304–0.305]
	1.664 [0.334]	1.665 [0.327]	1.672 [0.304]
Ag_ico_–H^a^	2.125–2.347 [0.058–0.097]	2.047–2.492 [0.052–0.103]	2.032–2.155 [0.070–0.103]
	2.270 [0.076]	2.234 [0.080]	2.108 [0.091]
H–Ir–H	93	95	-

a The three shortest Ag–H contacts per H atom are considered.

In the NMR spectra, the different metal nuclearities of the *nido*-IrH_2_Ag_11_ and *closo*-IrHAg_12_ units lead to significant variations in the shielding effects experienced by the hydrides. Notably, compared to the previously reported RhH_2_Ag_19_(dtp)_12_ and RhHAg_20_(dtp)_12_ systems,^18^ the chemical shift difference between species containing two hydrides and one hydride is more pronounced in the present case. In the ^1^H NMR spectrum of **1**, a hydride resonance is observed at −15.0 ppm, with an integration ratio of 4:17 relative to the ligands, in agreement with its composition. For **2**, the hydride signals split into two sets at −15.4 and −16.1 ppm with an integration ratio of 2:1, consistent with the presence of two inequivalent superatomic cores arising from its asymmetric structure. In **3**, a single hydride resonance appears at −16.1 ppm, with an integration ratio of 2:17 relative to the ligands, in agreement with its composition (Figure 3a). The ^31^P NMR spectra further support these structural features. The five ligands surrounding the central region of the cluster exhibit similar chemical environments. At the two termini of the rod-shaped core, each apex is capped by a ligand, and these two ligands share comparable chemical environments. In addition, the five ligands, which encircle each terminal region and form belt-like arrangements at both ends, display similar chemical environments within each set (Figure S3). As a result, the ligands can be grouped into three distinct phosphorus environments overall. However, due to the lower symmetry of the metal framework in **2**, the ten terminal ligands exhibit slightly differentiated chemical shifts, reflecting their inequivalent local environments (Figure 3b).

**Figure 3:**
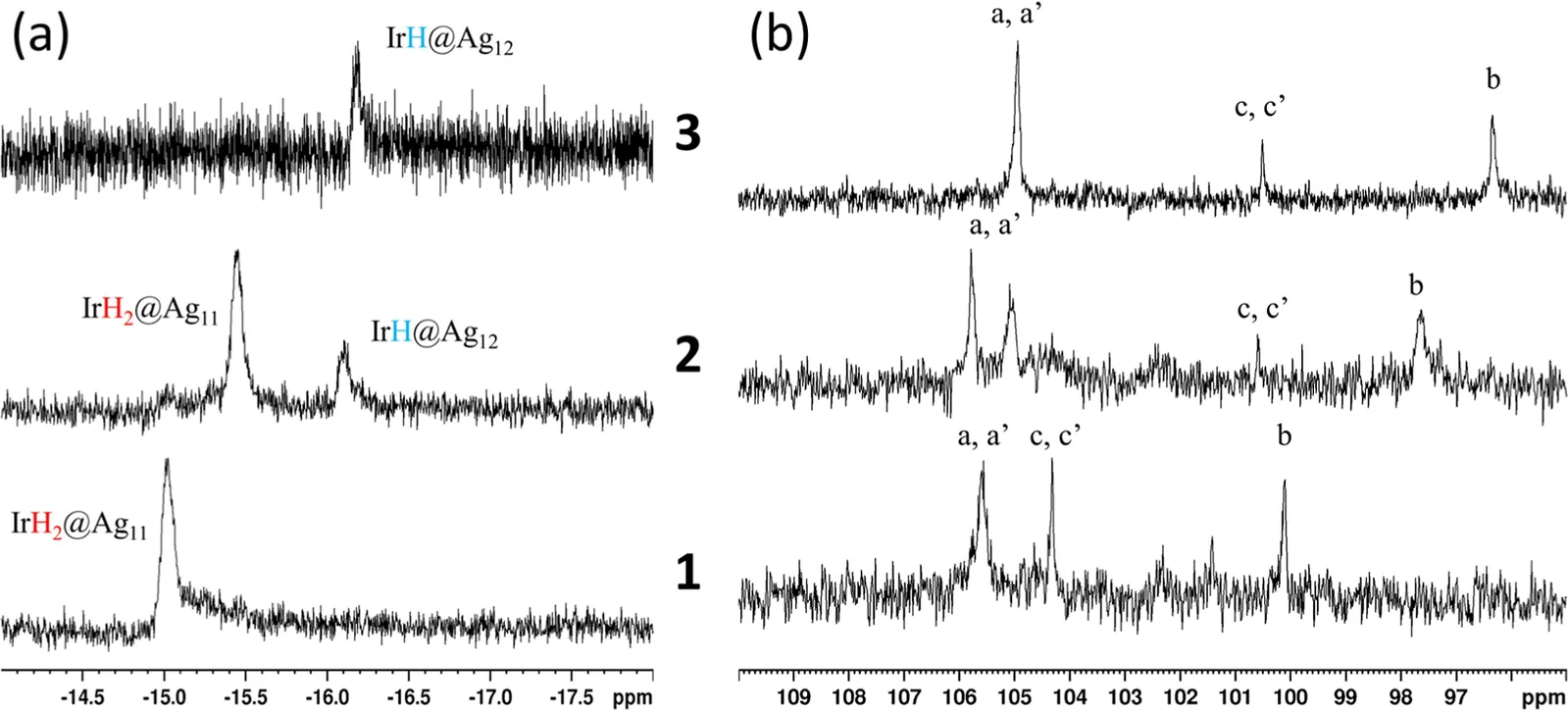
(a) ^1^H NMR spectrum (CDCl_3_) of **1**, **2**, and **3**. (b) ^31^P NMR spectrum (CDCl_3_) of **1**, **2**, and **3**.

In order to shed some light on the bonding of clusters **1**–**3**, DFT calculations at the BP86/Def2-TZVP level (see Computational Details in the Supporting Information) were performed on a simplified model in which the OPr groups of the ligands were replaced by hydrogen atoms, a simplification that has been validated in many previous investigations.^17,18,40,44,45,49,57^ Selected computed data are given in Tables 1 and 2. The optimized geometries of **1**, **2**, and **3** are in good agreement with their experimental X-ray counterparts (Table 1). In the *closo*-(IrH)@(Ag_12_) units of **2** and **3**, the hydride is connected to three Ag atoms and to the Ir atom. The corresponding distances and their associated Wiberg bond indices (Table 1) indicate that the hydride binds strongly with Ir and more weakly with Ag.^18,45^ A similar situation occurs in the *nido*-(IrH_2_)@(Ag_11_□) units of **1** and **2**, but this time the three Ag–H contacts are distributed nonhomogeneously into a shorter one (∼2.1 Å) and two longer ones (∼2.3 Å). Overall, the computed natural atomic orbital (NAO) charge distributions (Table 2) are quite similar to those found previously from their rhodium analogues^45^ and are consistent with the view of compounds **1**–**3** having a 16-electron core, isolobal to the van der Waals dimer Ne_2_, namely, [Ir_2_H_*x*_Ag_25–*x*_]^7+^ (*x* = 4, 3, 2 for **1**, **2**, and **3**, respectively). This core results from the assembly of two 8-electron [*closo*-(IrH)@(Ag_12_)]^4+^ and/or [*nido*-(IrH_2_)@(Ag_11_□)]^4+^ superatomic cores through vertex sharing (hence its charge 4 + 4 – 1 = +7), and it is stabilized by an outer shell composed of 17 dtp^–^ ligands and 10 capping Ag^+^ centers. The individual *closo* and *nido* icosahedral units have similar 1S^2^ 1P^6^ 1D^0^ electronic structures and interact similarly (and weakly) in the three clusters, giving rise to the highest occupied orbitals, which are weakly antibonding 1P combinations, and the lowest vacant orbitals, which are weakly bonding 1D combinations (Figures S8–S10). At this stage, it is important to remember that, like the Ag atoms, the encapsulated hydrogens donate their electron to the cluster *superatomic* count,^16–18,49,50^ thus somehow behaving as insertion dopants. On the other hand, their 1s AOs participate very little in the superatomic orbitals.^17,18,44,48,49^ In the three clusters, the NAO charges of the ten peripheral Ag_cap_ silvers (∼+0.6) are consistent with their Ag­(I) oxidation state (Table 2).^17,18,44,45,51,57^ Consistently, those of the mixed-valent icosahedral cores are lower (<+0.3), in particular that of the atom which is shared by the two icosahedral units (Ag_ico(shared)_) and those which occupy the end positions of the bis-icosahedral cores (Ag_ico(end)_). There is actually one exception, which concerns the two Ag_ico(S2)_ atoms, which, in each of the three compounds, are bonded to two sulfur atoms. Their NAO charges (∼+0.5) approach that of the outer Ag­(I) metal centers. This feature reinforces the idea suggested above that, in the *closo* cases, these Ag_ico(S2)_ atoms are more susceptible to leaving the metal framework as Ag^+^ ions upon adding additional encapsulated hydrides, thus generating *nido* units.

**Table 2:** Averaged NAO Charges Computed for Clusters **1**, **2**, and **3**

	(IrH_2_)_2_Ag_31_[S_2_P(OPr)_2_]_17_ (**1**)	(IrH_2_)(IrH)Ag_32_[S_2_P(OPr)_2_]_17_ (**2**)		(IrH)_2_Ag_33_[S_2_P(OPr)_2_]_17_ (**3**)
	2 × *nido* units	1 × *nido* unit	1 × *closo* unit	2 × *closo* unit
Ag_ico(shared)_	0.00	–0.02	–0.05	
Ag_ico(end)_	0.06	0.05	0.08	0.06
Ag_ico(S2)_	0.55	0.54	0.50	0.51
Ag_ico(regular)_	0.29	0.29	0.26	0.26
Ag_cap_	0.57	0.57	0.57	0.57
Ir	–1.13	–1.12	–1.27	–1.26
H	–0.22	–0.17	–0.27	–0.28

At this stage of the discussion, it is important to note that, unlike in the DFT-optimized structures, in the experimental structures, Ag_ico(S2)_ atoms exist only in *closo* icosahedra, i.e., two Ag_ico(S2)_ in **3** and one in **2**. This discrepancy is attributed to the use of simplified ligands in the computed models, which allow a more compact environment of the *closo* icosahedra. The Kohn–Sham frontier orbital diagrams of clusters **1**–**3** are shown in Figure S8–S10. Their highest occupied orbitals can be identified as the out-of-phase combinations of the 1P orbitals of their individual icosahedra, and their lowest vacant orbitals as the in-phase combinations of the icosahedra 1D orbitals. These electronic structure features are typical for 16-electron architecture made of two weakly interacting 8-electron icosahedral subunits, irrespective of their number of encapsulated hydrides.^36,38,42–45^ The time-dependent-DFT (TD-DFT)-simulated UV–vis spectra of clusters **1**–**3** are shown in Figure S11. They are in a satisfying agreement with their experimental counterparts (Figure 1d). In **1**–**3**, the low-energy shoulder of lowest energy (∼550–600 nm) can be identified as of 1P → 1D nature, with a large HOMO → LUMO contribution. The major band (∼400–450 nm) is also of 1P → 1D character, but with some 4d­(Ag_ico_) → 1D admixture.

## Conclusion

In summary, we report a series of iridium-doped silver-rich 16-electron clusters of the general formula Ir_2_H_*x*_Ag_35–*x*_[dtp]_17_ (*x* = 4, 3, 2), which have been thoroughly characterized. The three clusters have quasi-identical [Ag_10_(dtp)_17_]^7–^ passivating shells, and their 16-electron cores are made of two distorted vertex-sharing centered icosahedra. In the case of **1** (*x* = 4), the two icosahedra are *nido*-type (defective) (IrH_2_)@(Ag_11_□) units, whereas in the case of **3**, they are *closo*-type (IrH)@(Ag_12_) complete icosahedra. The unsymmetrical cluster **2** contains one *nido*-(IrH_2_)@(Ag_11_□) and one *closo*-(IrH)@(Ag_12_) distorted icosahedron. Formally, comparison of the *closo*-(IrH)@(Ag_12_) and *nido*-(IrH_2_)@(Ag_11_□) units shows that accommodation of a second hydride is associated with the absence of one core Ag atom rather than one Ag atom from the passivating layer. This unexpected feature is distinct from previously reported hydride-containing superatomic systems in which a peripheral capping silver atom is removed on formal hydride addition. This peculiarity is likely to be related to the specific coordination environment of one of the silver atoms of the *closo*-(IrH)@(Ag_12_) units. These findings not only establish a new type of superatomic building unit, but also expand the structural diversity of superatomic assemblies. From a more conceptual perspective, clusters **1**–**3** demonstrate the *closo*-to-*nido* isoelectronic relationship in 8-electron icosahedral superatomic units. In contrast, it has been shown that when three or four vertices are missing from the icosahedron, generating largely defective structures, the favored electron counts are reduced to six (three vacancies) and four (four vacancies), respectively.^47,49–52^ We are currently investigating the electronic relationships between these nonisoelectronic architectures, as well as the possibility of generating hydride-containing *arachno*-type species (2-vacancy icosahedral structures).

## Experimental Section

### General Remarks

All chemicals were purchased from commercial sources and used as received. Solvents were purified following standard protocols. All reactions were carried out under a N_2_ atmosphere by using standard Schlenk techniques. [Ag­(CH_3_CN)_4_]­PF_6_ and NH_4_[S_2_P­(O^*n*^Pr)_2_] were prepared by following the procedure reported in literature.^58,59^ These reagents are considered corrosive and should be used with caution. NMR spectra were recorded on a Bruker AVII-400 and AV-600 MHz NMR spectrometers. The chemical shift (δ) is reported in ppm and Hz, respectively. ESI-mass spectra were recorded on a Bruker maXis Q-TOF mass spectrometer (Bruker Daltonik GmbH, Germany). UV–visible–NIR absorption spectra were measured on a Shimadzu UV3101PC spectrophotometer at 298 K, using quartz cells with a path length of 1 cm.

### Synthesis

#### Synthesis of (IrH_2_)_2_Ag_31_[S_2_P­(OPr)_2_]_17_ (**1**), (IrH)­(IrH_2_)­Ag_32_[S_2_P­(OPr)_2_]_17_ (**2**), and (IrH)_2_Ag_33_[S_2_P­(OPr)_2_]_17_ (**3**)

In a Schlenk tube, [Ag­(CH_3_CN)_4_]­PF_6_ (0.47 g, 1.12 mmol) and NH_4_[S_2_P­(O^*n*^Pr)_2_] (0.14 g, 0.60 mmol) were dissolved in THF and stirred at −20 °C for 5 min. [Ir­(COD)­Cl]_2_ (0.03 g, 0.07 mmol) was then added to the above solution. After 10 min, NaBH_4_ (0.04 g, 1.12 mmol) was added, and the mixture was stirred for 12 h. The reaction mixture was dried under reduced pressure. The residue was washed with DCM/DI–water. The DCM layer was dried under reduced pressure and subsequently purified over Al_2_O_3_ using ether/DCM = 1/4 as the eluent. The resulting gray product was further separated by TLC using ether/hexane = 1/4 as the mobile phase. Compound **1** exhibited the highest *R*_f_, followed by **2** and **3**. The purified products were recrystallized from MeOH at room temperature over 3–4 days to afford black crystals suitable for SCXRD. The isolated crystals of **1**–**3** remained stable under ambient conditions for at least three months without obvious deterioration and remained suitable for structural characterization.

#### Synthesis of the Deuterated Analogues **1**_**D**_, **2**_**D**_, and **3**_**D**_

Compounds **1**_**D**_, **2**_**D**_, and **3**_**D**_ were prepared following the same synthetic and purification procedures as those used for **1–3**, except that NaBD_4_ was used in place of NaBH_4_.

#### (IrH_2_)_2_Ag_31_[S_2_P­(OPr)_2_]_17_ (**1**) (2.7 mg; Yield: 1% Based on Ag)

^31^P­{^1^H} NMR (161.97 MHz, CDCl_3_, δ, ppm, r.t.): 105.6, 104.3, 100.1 ^1^H NMR (400 MHz, CDCl_3_, δ, ppm, r.t.): −15.0 (br, H, 4H) 0.8–1.0 (m, CH_3_, 102H), 1.67–1.87 (m, CH_2_, 68H), 4.05–4.36 (m, CH_2_, 68H). ESI-MS (*m*/*z*): exp. 7465.1924 (calcd for [M + Ag]^+^, 7465.2022) UV–vis (λ_max_ in nm, ε in M^–1^ cm^–1^): 453 (40,000) 609 (8000).

#### (IrH)­(IrH_2_)­Ag_32_[S_2_P­(OPr)_2_]_17_ (**2**) (1.6 mg; Yield: 0.5% Based on Ag)

^31^P­{^1^H} NMR (161.97 MHz, CDCl_3_, δ, ppm, r.t.): 105.3, 98.7. ^1^H NMR (400 MHz, CDCl_3_, δ, ppm, r.t.): −16.1 (br, H, 1H) −15.5 (br, H, 2H) 0 0.8–1.0 (m, CH_3_, 102H), 1.67–1.87 (m, CH_2_, 68H), 4.05–4.36 (m, CH_2_, 68H). ESI-MS (*m*/*z*): exp. 7571.0521 (calcd for [M + Ag]^+^, 7571.1010) UV–vis (λ_max_ in nm, ε in M^–1^ cm^–1^): 386 (52,000) 470 (38,000) 630 (6000).

#### (IrH)_2_Ag_33_[S_2_P­(OPr)_2_]_17_ (**3**) (2.6 mg; Yield: 1% Based on Ag)

^31^P­{^1^H} NMR (161.97 MHz, CDCl_3_, δ, ppm, r.t.): 105.6, 104.3, 100.1 ^1^H NMR (400 MHz, CDCl_3_, δ, ppm, r.t.): −16.2 (br, H, 2H) 0.8–1.0 (m, CH_3_, 102H), 1.67–1.87 (m, CH_2_, 68H), 4.05–4.36 (m, CH_2_, 68H). ESI-MS (*m*/*z*): exp. 7678.9547 (calcd for [M + Ag]^+^, 7678.9988) UV–vis (λ_max_ in nm, ε in M^–1^ cm^–1^): 386 (50,000) 472 (34,000) 652 (6000).

### X-ray Crystallography

Single-crystal X-ray diffraction data were collected using an XtaLAB Synergy, single source at home/near, HyPix-Arc 100 diffractometer operating at *T* = 100.01(10) K. Data were measured using w scans of 0.5° per frame for 0.1 s using Cu Kα radiation. The diffraction pattern was indexed, and the total number of runs and images was determined based on the strategy calculation in the CrysAlisPro program (Rigaku, Version 1.171.44.120a, 2025). Data reduction, scaling, and absorption corrections were performed using CrysAlisPro (Rigaku, V1.171.44.120a, 2025). The structure was solved with the ShelXT (Sheldrick, 2015) structure solution program using the intrinsic phasing solution method and by using Olex2 as the graphical interface.^60,61^ Further details are provided in Table S2. CCDC 2552810–2552812 contain the supplementary crystallographic data for compounds **1** to **3** in this article. These data can be obtained free of charge from the Cambridge Crystallographic Data Centre via http://www.ccdc.cam.ac.uk/data_request/cif.

## Computational Details

Geometry optimizations were performed by density functional theory (DFT) calculations with the Gaussian 16 package,^62^ using the BP86 functional^63,64^ and the all-electron Def2-TZVP set from the EMSL Basis Set. Exchange library.^65,66^ All of the optimized geometries were characterized as true minima by vibrational analysis. The NAO charges and Wiberg bond indices were computed with the NBO 6.0 program^67,68^ on single-point calculations performed with the BP86 functional and the Def2-SVP basis set for computational limitations.^69,70^ The UV–visible transitions were calculated by means of time-dependent DFT (TD-DFT) calculations, with the CAM-B3LYP functional^71^ and the Def2-TZVP basis set. Only singlet–singlet, i.e., spin-allowed, transitions have been computed. The UV–visible spectra were simulated from the computed TD-DFT transitions and their oscillator strengths by using the Multiwfn program;^72^ each transition is associated with a Gaussian function of half-height width equal to 2000 cm^–1^. The compositions of the molecular orbitals were calculated by using the AOMix program.^73^

## Supplementary Material



## Notes

Characterization data, structural figures, crystallographic tables, and DFT computational results (PDF)
