# Supplementary material for: Emergence of Nido-Icosahedral Superatomic Units in Iridium-Doped Silver Cluster Assemblies
Source: Inorg Chem. 2026 Aug 17;65(34):20020–8. doi: 10.1021/acs.inorgchem.6c03151 (PMC13536885; doi:10.1021/acs.inorgchem.6c03151)
Supplement: Supplementary file 1 [file ic6c03151_si_001.pdf]

Supplementary Information

Emergence of *Nido*-Icosahedral Superatomic units in Iridium-Doped Silver Cluster Assemblies

Tzu-Hao Chiu,[a] Michael N. Pillay,[a] Jose V. Rival,[b] Yoshiki Niihori,[c] Yuichi Negishi,[b] Samia Kahlal,[d] Jean-Yves Saillard,[d]* and C. W. Liu[a]*

[a] Department of Chemistry, National Dong Hwa University, Hualien 97401, Taiwan (Republic of China)

[b] Institute of Multidisciplinary Research for Advanced Materials, Tohoku University, Katahira 2-1-1, Aoba-ku, Sendai 980-8577, Japan

[c] Faculty of Textile Science and Technology, Shinshu University, Tokida, Ueda, Nagano 386-8567, Japan

[d] Univ Rennes, CNRS, ISCR-UMR 6226, F-35000 Rennes, France

[*] Correspondence: E-mail: chenwei@gms.ndhu.edu.tw; Tel: +886-3-890-3607; Fax : +886-3-890-0162; http://faculty.ndhu.edu.tw/~cwl/index.htm

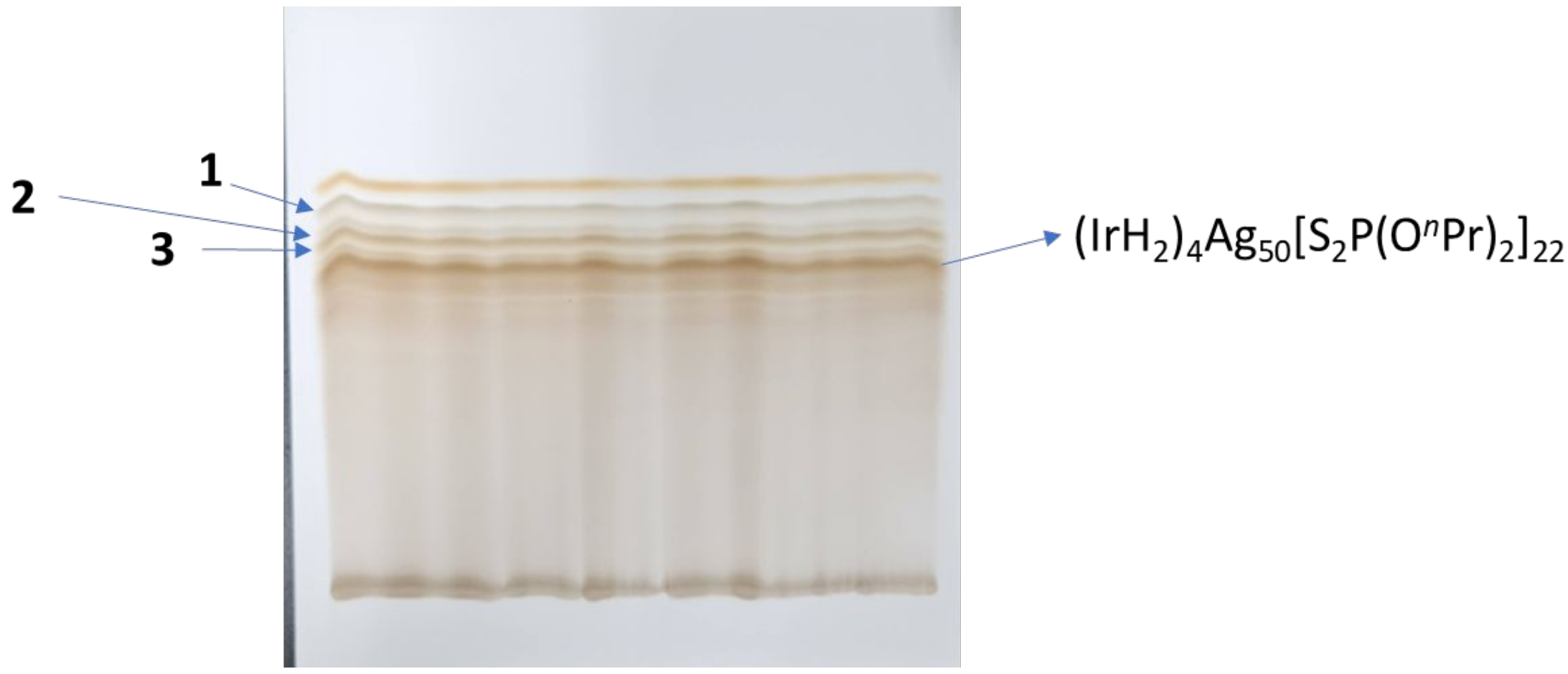

Figure S1. Photograph of the preparative TLC separation using ether/hexane (1:4) as the mobile phase. The additional band corresponds to the previously reported Ir_4Ag_{50} cluster. (*J. Am. Chem. Soc.* **2026***, 148,* 22481–22487.)

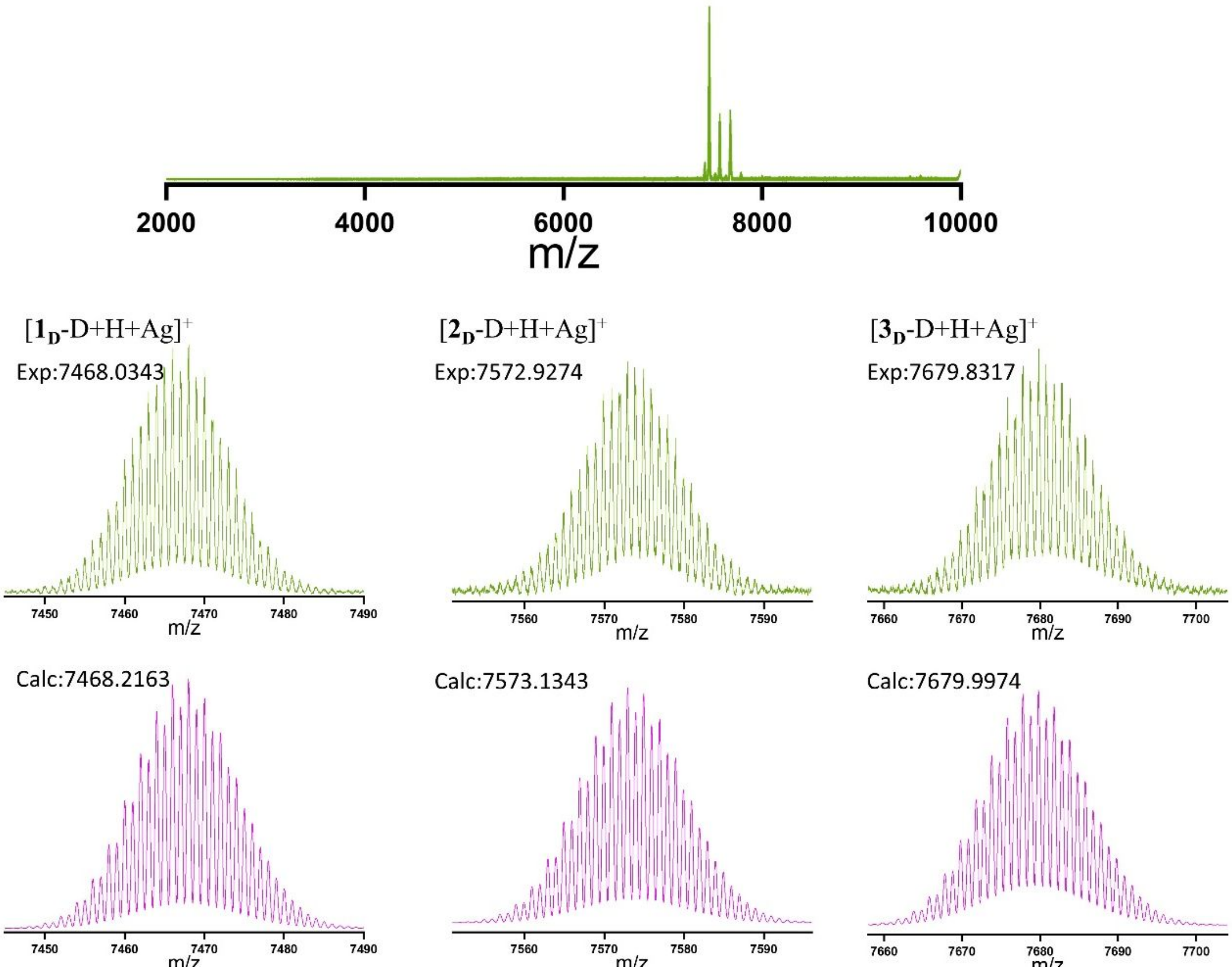

Figure S2. Positive-mode ESI mass spectrum of $\mathbf{1_D}$, $\mathbf{2_D}$ and $\mathbf{3_D}$ (top).

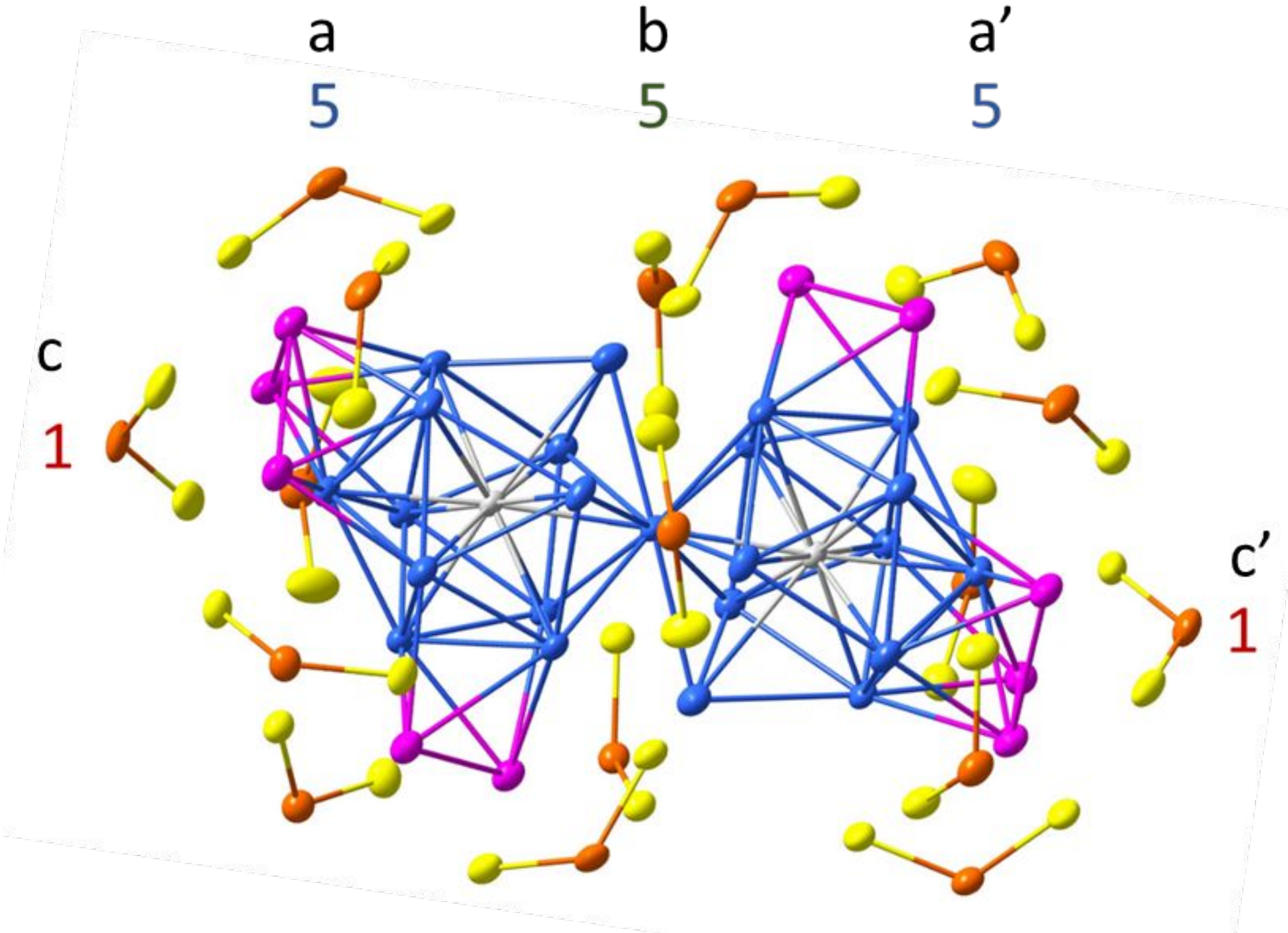

Figure S3. Ligand arrangement observed in the solid state.

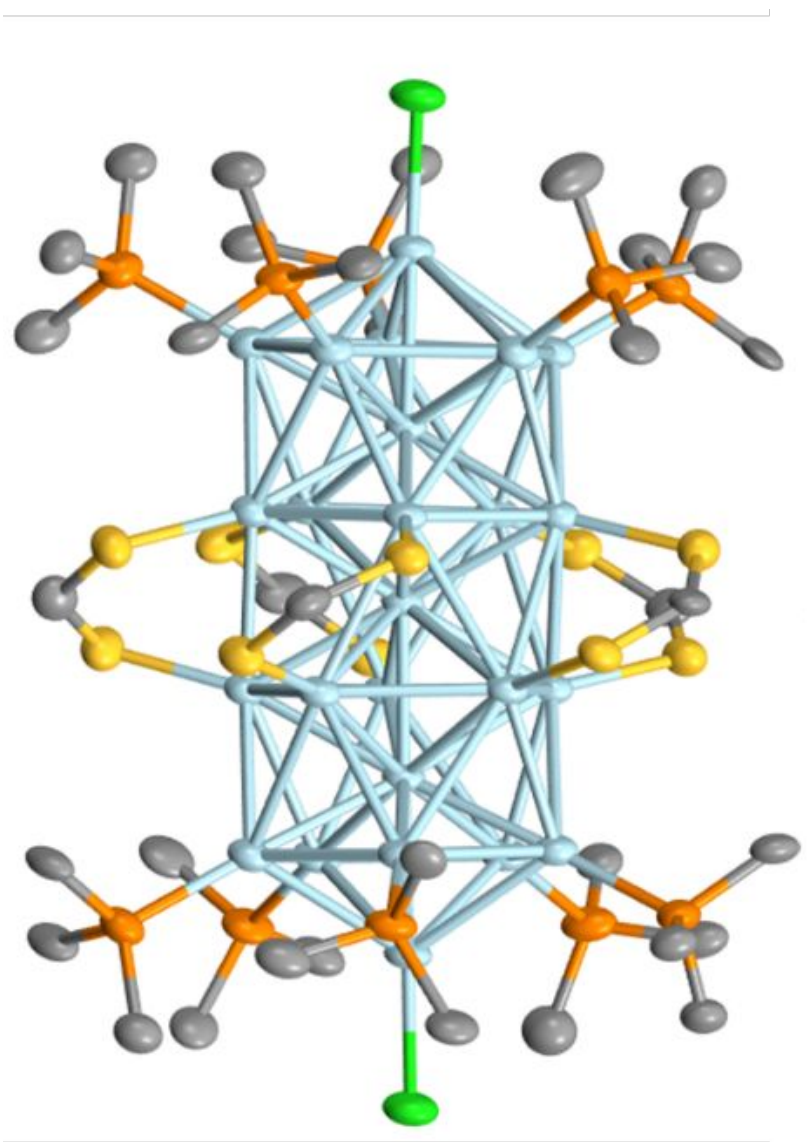

$[Au_{25}Cl_2(dtc)_5(PPh_3)_{10}]^{2+}$

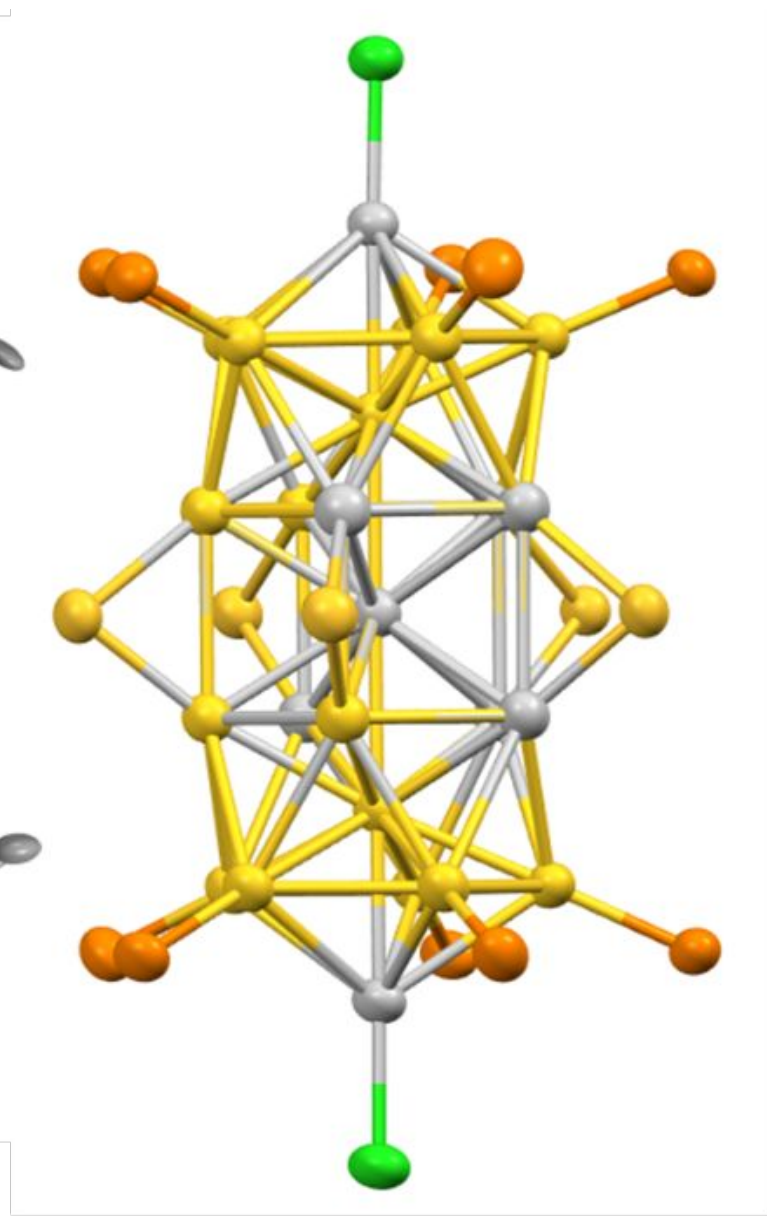

$[(AuAg)_{25}(SR)_5Cl_2(PPh_3)_{10}]^{2+}$

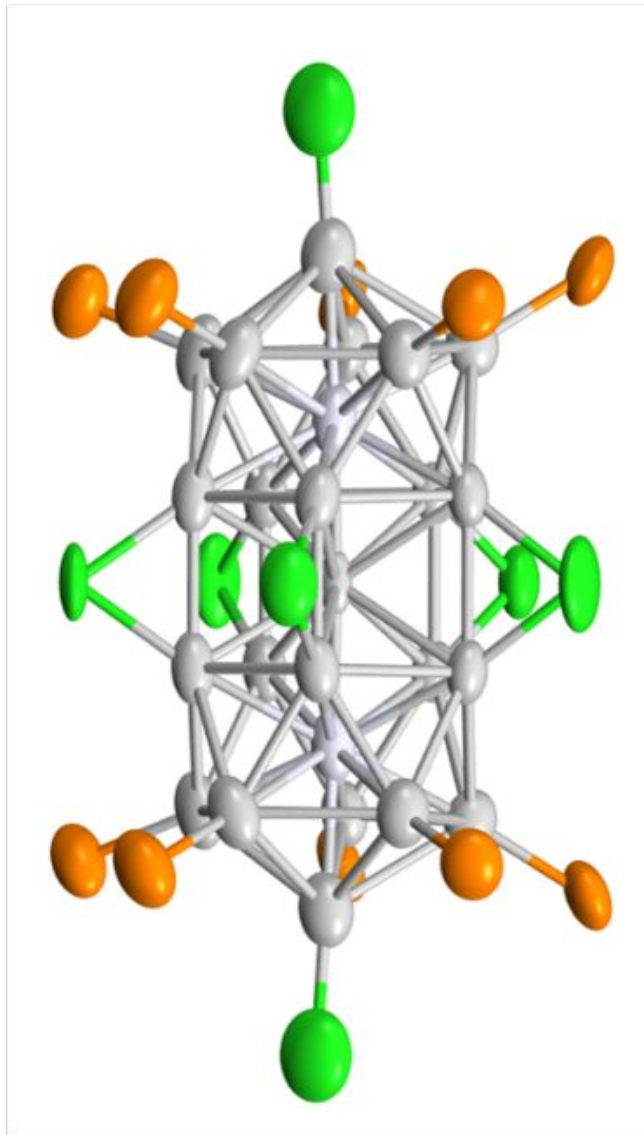

$Pt_2Ag_{23}Cl_7(PPh_3)_{10}$

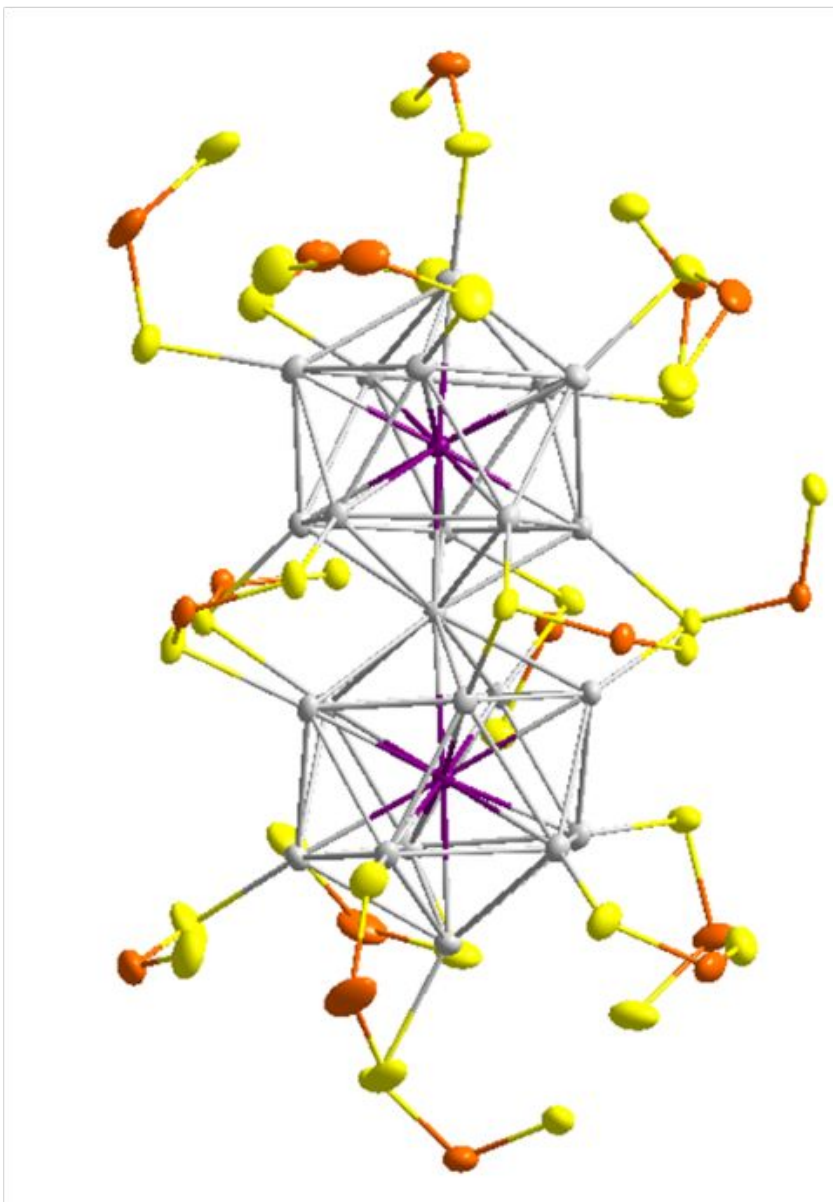

$Pt_2Ag_{33}(dtp)_{17}$

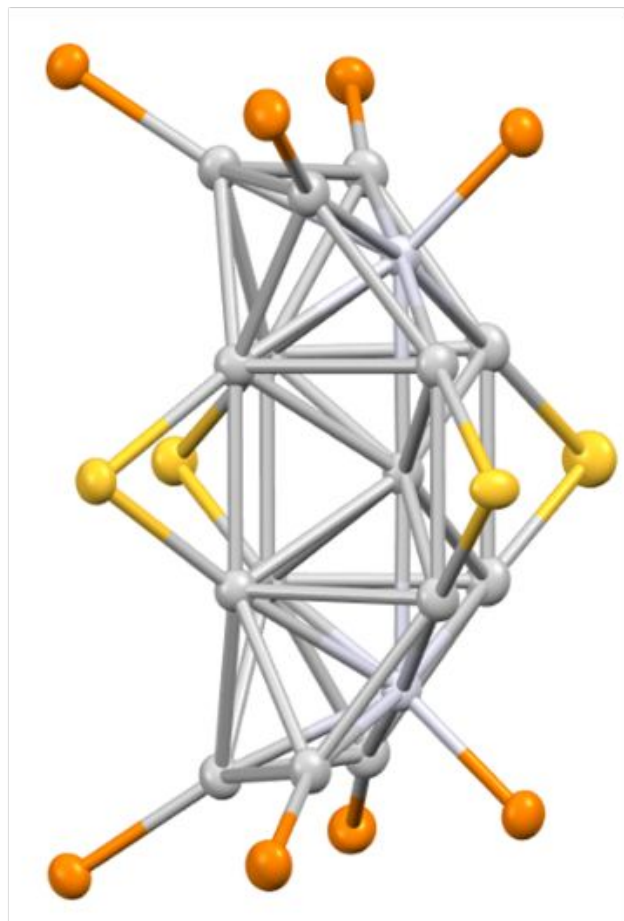

$[Pt_2Ag_{15}(SR)_4(DPPOE)_4H]^{2+}$

Figure S4. Representative examples of 16-electron bi-icosahedral silver clusters and related structures, including a vertex-sharing assembly of two incomplete icosahedral units. (dtc: $[S_2CNR_2]^-$; dtp: $[S_2P(OR)_2]^-$; DPPOE: 1,2-Bis(diphenylphosphino)ethane)

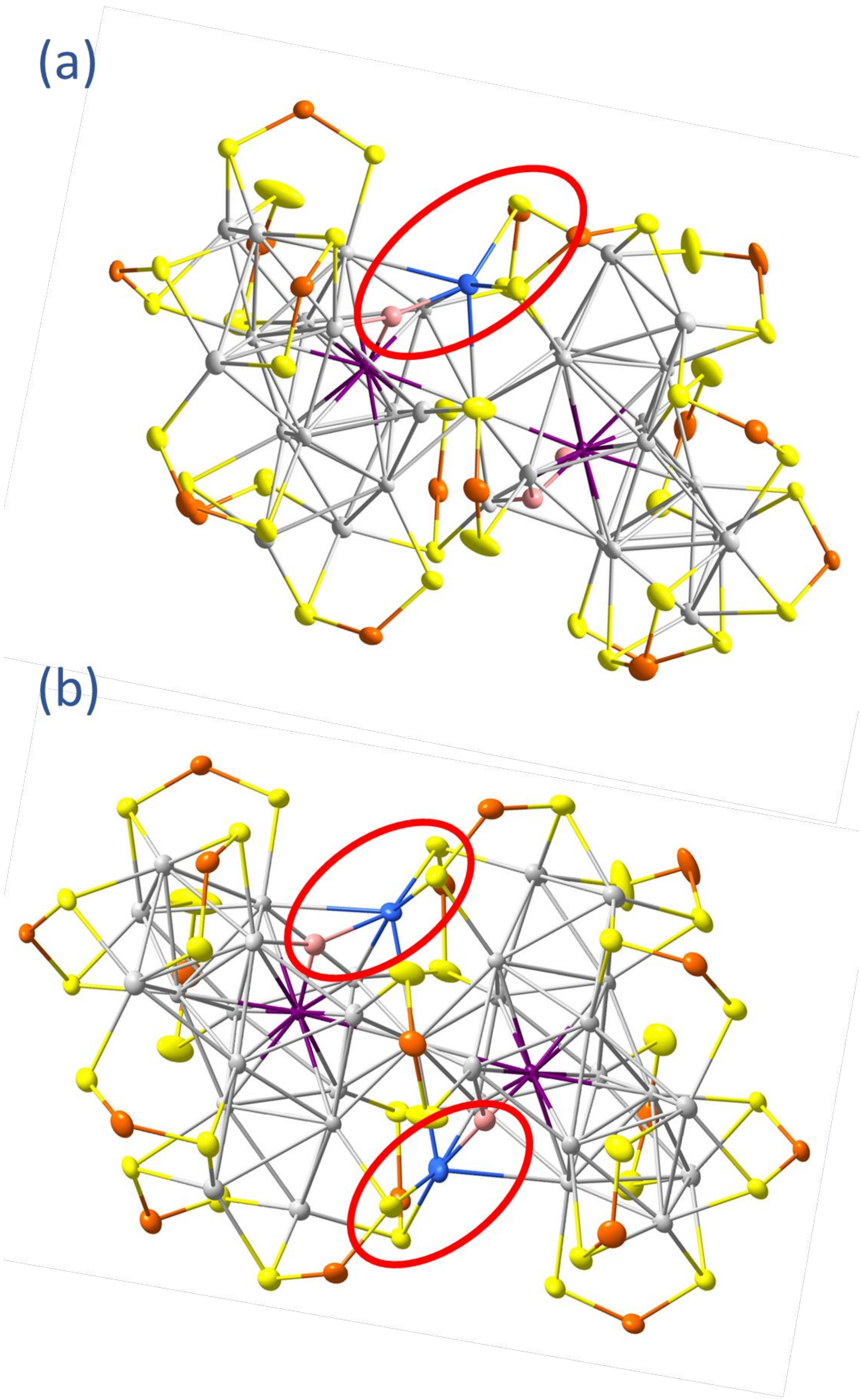

Figure S5. Total structure of (a) **2** (b) **3**, highlighting the unusual Ag coordination site bound to two S atoms and one H. Color code: Ir (purple), Ag (gray, blue), S (yellow), P (orange), H (pink)

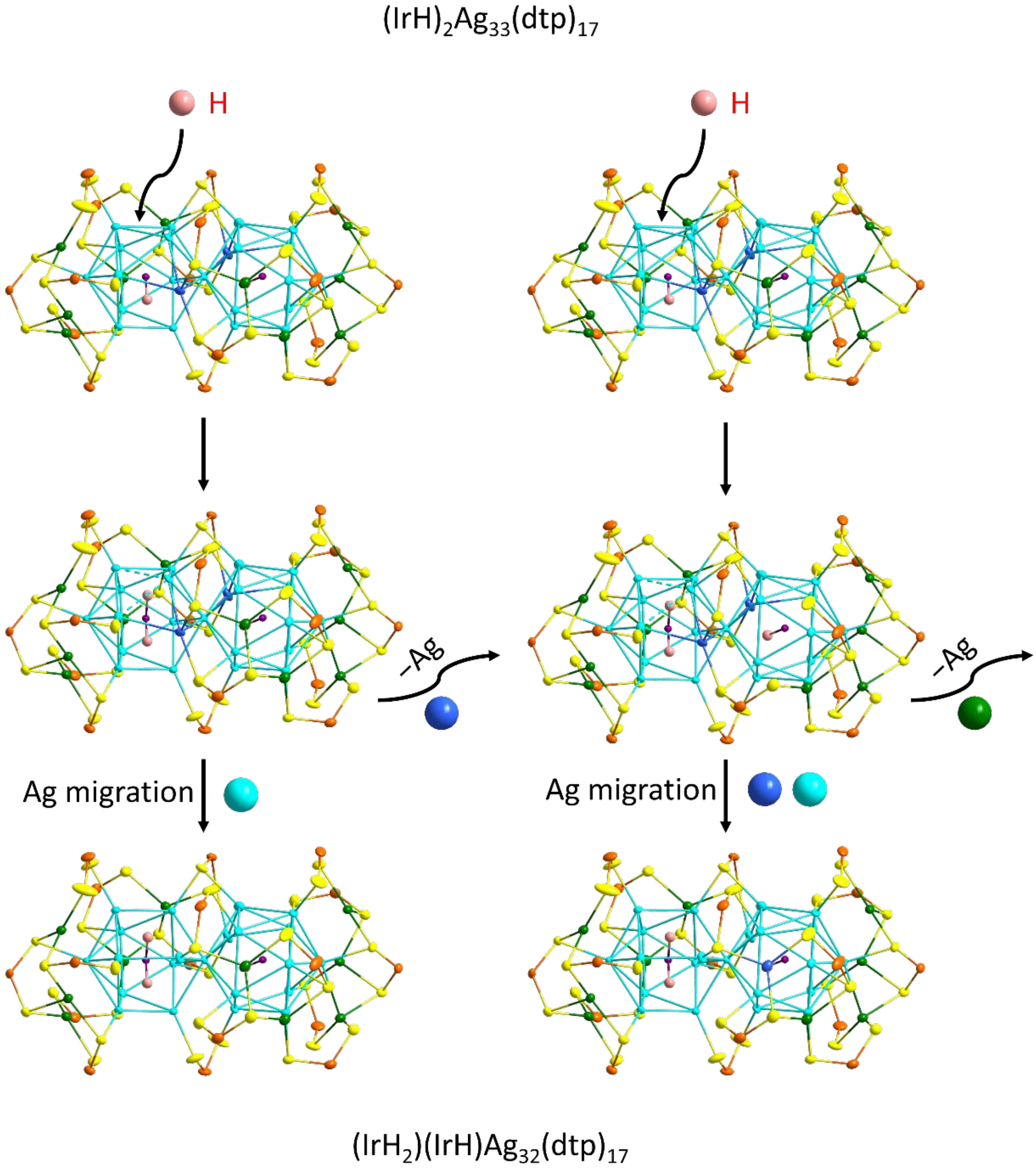

Figure S6. Plausible pathways accounting for the formation of the nido-type unit. Color code: Ir (purple), Ag_{ico} (sky blue), $Ag_{ico(S2)}$ (blue), Ag_{cap} (green), S (yellow), P (orange), H (pink)

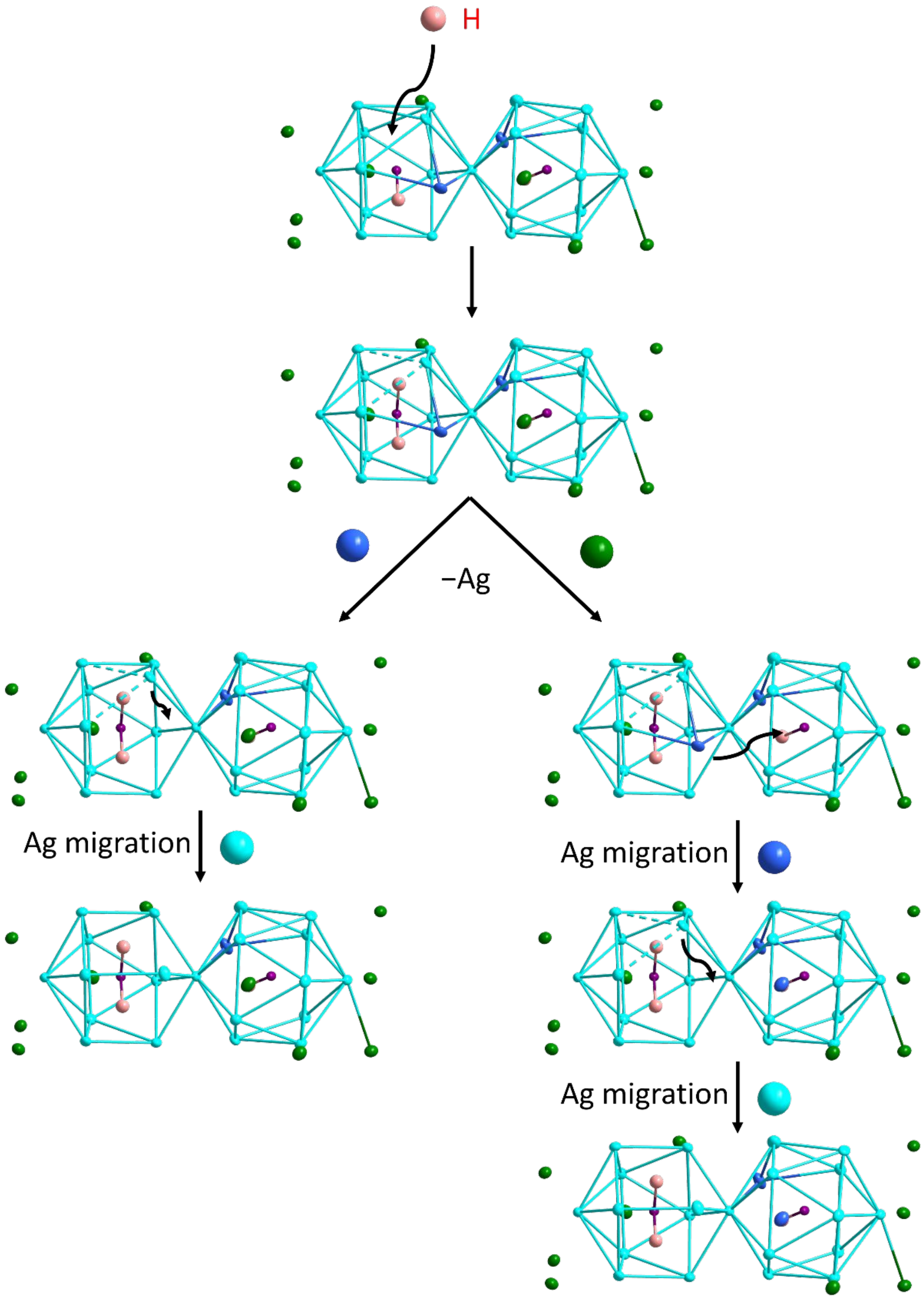

Figure S7. Plausible pathways accounting for the formation of the nido-type unit. Ligands are omitted for clarity. Color code: Ir (purple), Ag_{ico} (sky blue), $Ag_{ico(S2)}$ (blue), Ag_{cap} (green), H (pink)

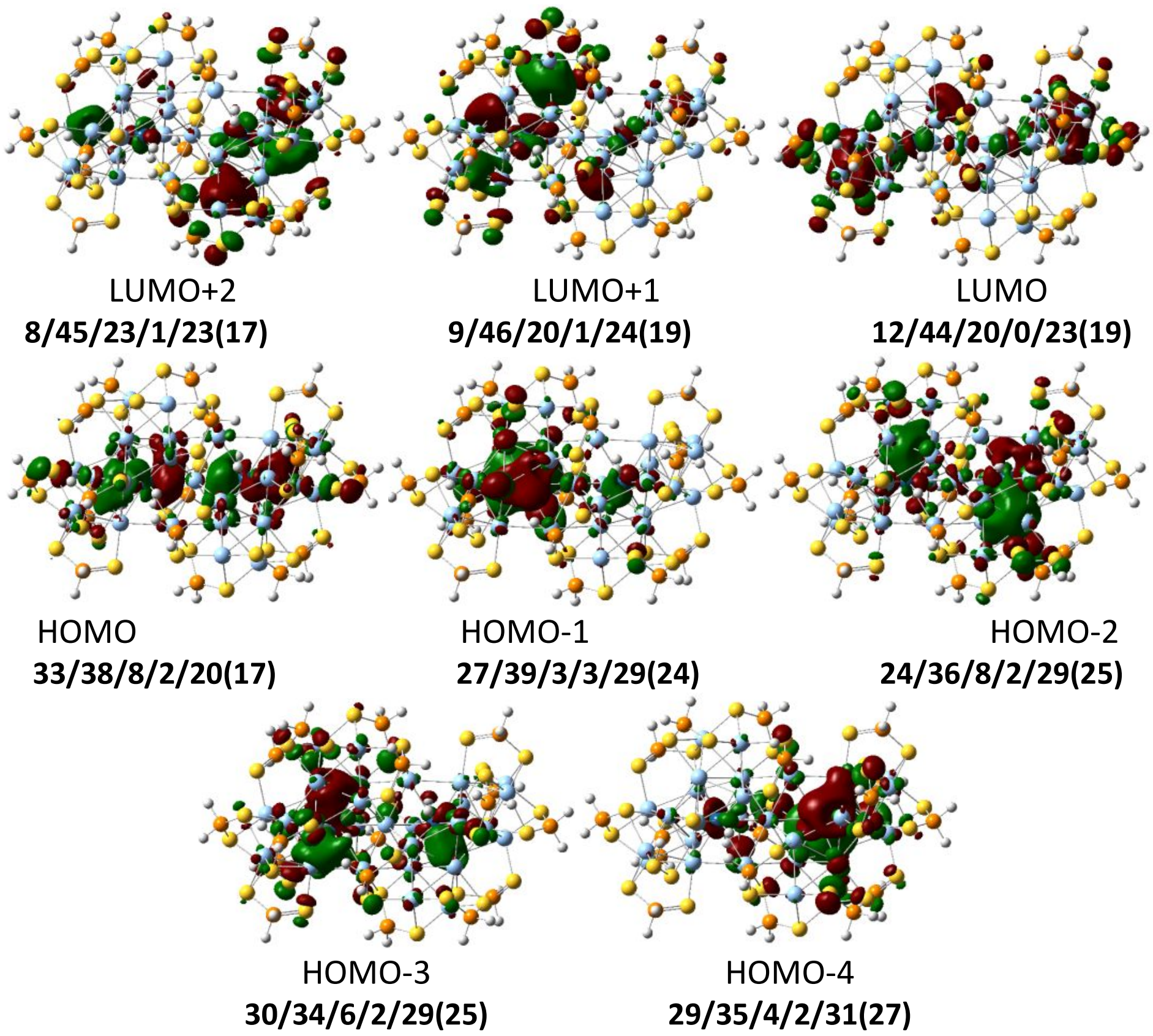

1

Figure S8. Kohn-Sham frontier orbitals of **1**. Atomic contributions (in %) are given in the order: Ir/Ag_{ico}/Ag_{cap}/H/ligand(S).

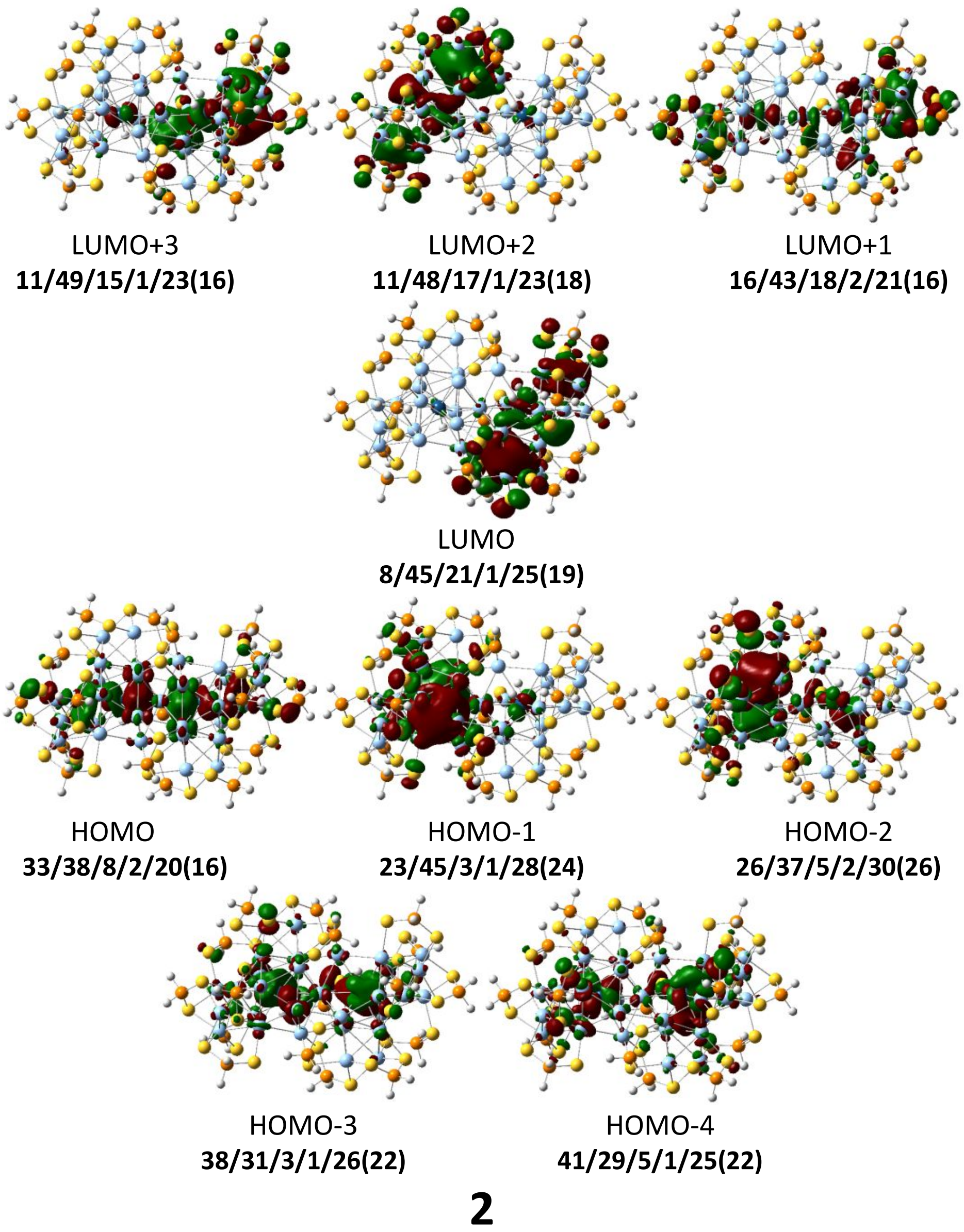

Figure S9. Kohn-Sham frontier orbitals of **2**. Atomic contributions (in %) are given in the order: Ir/Ag_{ico}/Ag_{cap}/H/ligand(S).

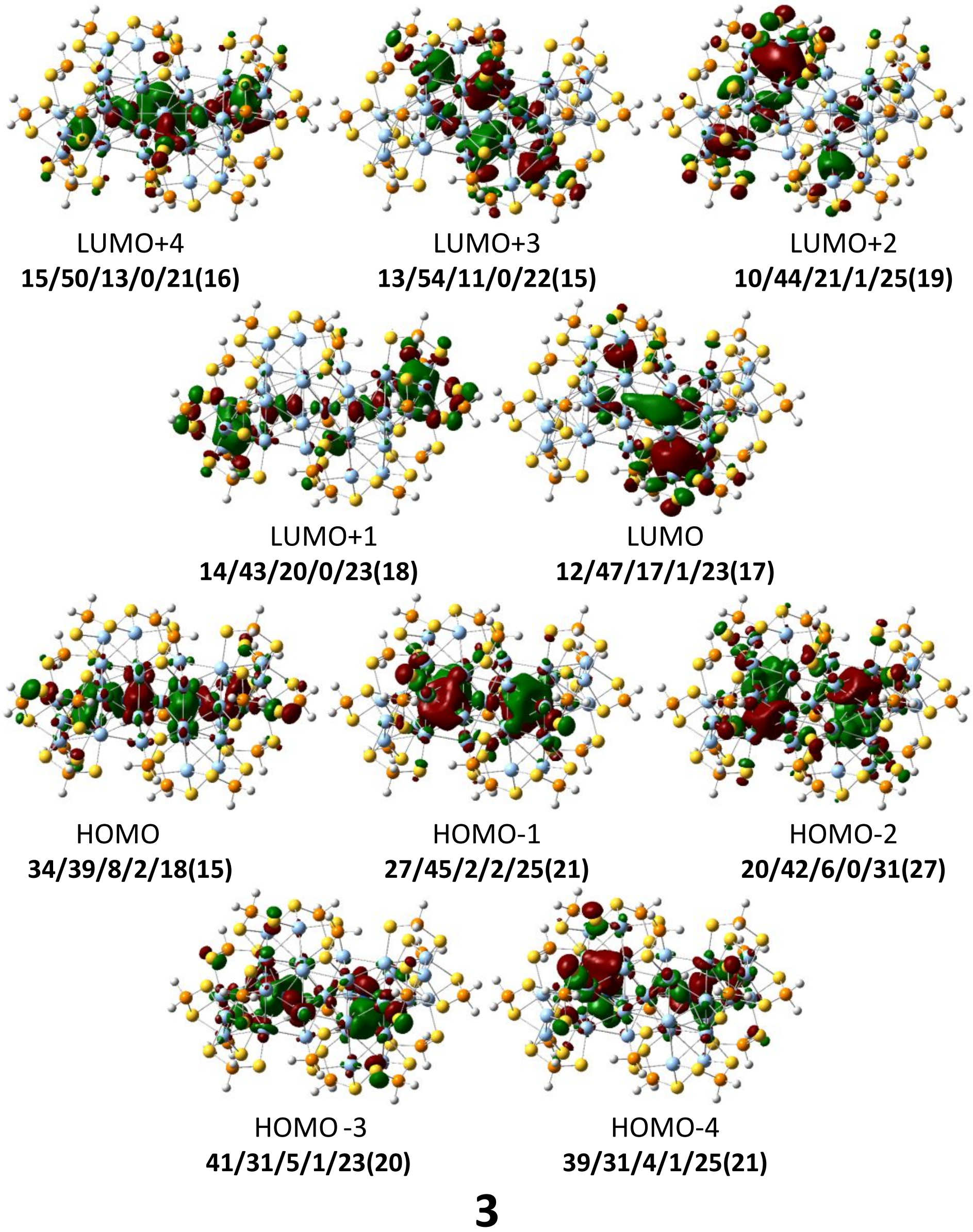

Figure S10. Kohn-Sham frontier orbitals of **3**. Atomic contributions (in %) are given in the order: Ir/Ag_{ico}/Ag_{cap}/H/ligand(S).

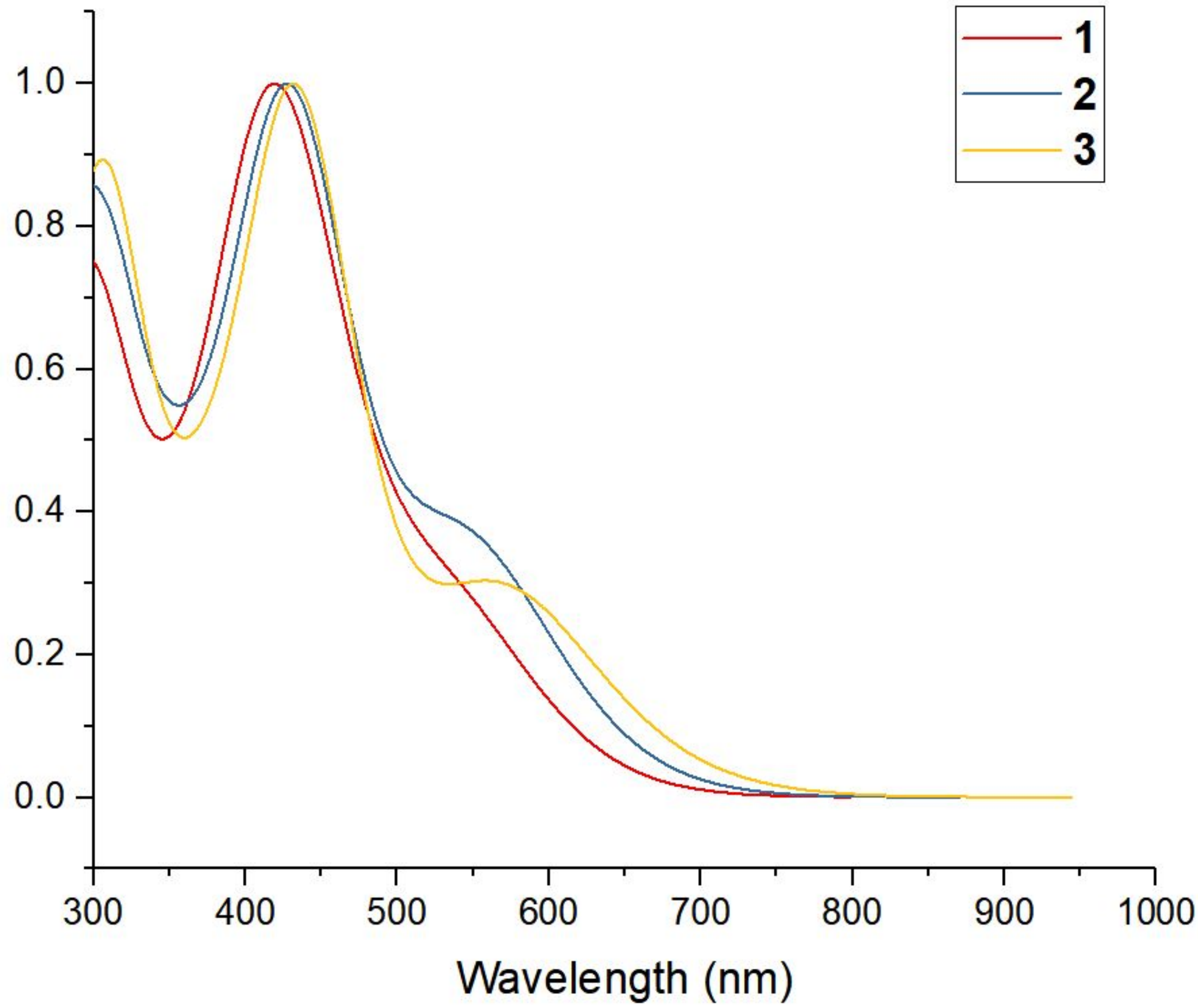

Figure S11. TD-DFT-simulated UV-vis absorption spectra of **1**, **2** and **3**.

Table S1. Selected X-ray crystallographic data of **1**, **2** and **3**

Compound	**1**	**2**	**3**
CCDC no.	2552810	2552811	2552812
Chemical formula	$C_{102}H_{238}Ag_{31}Ir_2O_{34}P_{17}S_{34}$	$C_{102}H_{238}Ag_{32}Ir_2O_{34}P_{17}S_{34}$	$C_{204}H_{477}Ag_{66}Ir_4O_{68}P_{34}S_{68}$
Formula weight	7353.81	7461.68	7570.06
Wavelength, Å	0.71073	1.54184	1.54184
Crystal System	Triclinic	Monoclinic	Triclinic
Space group	$P\bar{1}$	$C2/c$	$P\bar{1}$
a, Å	20.3081(16)	23.9179(3)	16.25780(10)
b, Å	33.265(3)	29.4746(5)	34.2992(2)
c, Å	34.012(3)	29.8129(4)	38.9110(3)
α, deg.	72.985(2)	90	72.9830(10)
β, deg.	78.1505(18)	100.3750(10)	85.1600(10)
γ, deg.	75.603(2)	90	83.6340(10)
V, $Å^3$	21062(3)	20673.6(5)	20589.2(3)
Z	4	4	4
Temperature, K	100(2)	100(2)	100(2)
ρ_{calcd}, g/cm^3	2.319	2.397	2.442
μ, mm^{-1}	4.582	30.992	31.861
θ_{max}, deg.	25.000	139.994	140
Completeness, %	98.9	99.9	99.2
Reflection collected / unique	138911 / 73336 [R(int) = 0.0533]	162537 / 19593 [R(int) = 0.1958]	379450 / 77441 [R(int) = 0.0989]
Restraints / parameters	1150 / 4040	825/1071	2876/4156
$^aR1$, $^bwR2$ [I > 2σ(I)]	0.0672, 0.1564	0.0947, 0.2748	0.0720, 0.1892
$^aR1$, $^bwR2$ (all data)	0.1076, 0.1839	0.1082, 0.2919	0.0801, 0.1956
GOF	1.058	1.052	1.045
Largest diff. peak and hole, e/$Å^3$	3.772 and -2.447	3.02/-1.75	4.54/-4.93

$^a R1 = \Sigma \,|\, |F_o| - |Fc| \,|\, / \Sigma |F_o|$. $^b wR2 = \{\Sigma[w(F_o^2 - F_c^2)^2]/\Sigma[w(F_o^2)^2]\}^{1/2}$.

Table S2. Cartesian coordinates of the DFT-optimized structures of **1**, **2** and **3**.

1

Ir	-0.017526	2.859919	0.079231
Ir	0.017526	-2.859919	0.079231
H	-0.749515	2.387668	1.490504
H	-1.353235	2.497901	-0.849681
H	1.353235	-2.497901	-0.849681
H	0.749515	-2.387668	1.490504
Ag	0.350088	5.704162	0.172739
Ag	-0.002027	4.169805	2.637777
Ag	2.498611	3.893059	1.066658
Ag	1.279424	1.438057	2.191373
Ag	4.722166	3.552059	-0.942787
Ag	-2.329530	4.369766	0.639674
Ag	1.764983	3.974410	-1.886279
Ag	-1.089450	4.400887	-2.109993
Ag	2.385519	1.456690	-0.666074
Ag	-0.063942	1.622085	-2.515156
Ag	0.000000	0.000000	-0.027100
Ag	-1.646957	6.991743	2.035867
Ag	-2.558033	1.351867	0.472648
Ag	-2.222433	6.964766	-1.132701
Ag	2.558033	-1.351867	0.472648
Ag	-2.385519	-1.456690	-0.666074
Ag	-1.279424	-1.438057	2.191373
Ag	4.335159	1.433787	1.781436
Ag	2.329530	-4.369766	0.639674
Ag	-1.764983	-3.974410	-1.886279
Ag	-2.498611	-3.893059	1.066658
Ag	0.063942	-1.622085	-2.515156
Ag	0.002027	-4.169805	2.637777
Ag	1.089450	-4.400887	-2.109993
Ag	-0.350088	-5.704162	0.172739
Ag	0.810733	6.822389	-2.581287
Ag	-4.335159	-1.433787	1.781436
Ag	-4.722166	-3.552059	-0.942787
Ag	2.222433	-6.964766	-1.132701
Ag	1.646957	-6.991743	2.035867

Ag	-0.810733	-6.822389	-2.581287
S	0.682480	8.073542	1.424813
S	0.687160	4.950927	4.953728
S	4.669578	5.528306	0.798560
S	1.944798	0.532350	4.478043
S	3.953554	4.049528	-3.381541
S	6.262021	1.642229	-0.065906
S	-4.845720	4.785466	0.489095
S	-3.121552	5.661073	-3.263692
S	3.904290	-0.388034	-1.769873
S	-1.438281	0.983381	-4.570365
S	-3.689216	8.103186	0.725866
S	-2.590795	5.893109	4.156715
S	-0.575975	8.959777	-1.795647
S	-4.267017	1.127436	2.415352
S	4.422761	3.350128	3.523899
S	4.267017	-1.127436	2.415352
S	-3.904290	0.388034	-1.769873
S	-1.944798	-0.532350	4.478043
S	3.322590	7.277927	-2.112967
S	0.000000	5.879762	-4.862249
S	1.438281	-0.983381	-4.570365
S	4.845720	-4.785466	0.489095
S	-3.953554	-4.049528	-3.381541
S	-4.669578	-5.528306	0.798560
S	-0.687160	-4.950927	4.953728
S	3.121552	-5.661073	-3.263692
S	-0.682480	-8.073542	1.424813
S	-6.262021	-1.642229	-0.065906
S	-4.422761	-3.350128	3.523899
S	3.689216	-8.103186	0.725866
S	-3.322590	-7.277927	-2.112967
S	2.590795	-5.893109	4.156715
S	0.575975	-8.959777	-1.795647
S	0.000000	-5.879762	-4.862249
P	0.274555	9.440706	-0.016292
P	-0.997212	5.949129	5.405391
P	5.462404	4.737366	2.504628

P	2.953008	-1.123111	3.993091
P	4.160203	6.083790	-3.482044
P	5.795924	0.002849	-1.177205
P	-5.034062	6.642975	1.212572
P	-1.954438	6.337501	-4.794046
P	0.000000	0.000000	-5.582202
P	-2.953008	1.123111	3.993091
P	-5.795924	-0.002849	-1.177205
P	5.034062	-6.642975	1.212572
P	-4.160203	-6.083790	-3.482044
P	-5.462404	-4.737366	2.504628
P	0.997212	-5.949129	5.405391
P	1.954438	-6.337501	-4.794046
P	-0.274555	-9.440706	-0.016292
H	1.491261	10.116769	-0.290314
H	-0.503774	10.483963	0.549855
H	-1.479694	5.527530	6.675183
H	-0.678402	7.303152	5.702292
H	6.786102	4.294038	2.232897
H	5.726324	5.829665	3.372561
H	2.093484	-2.241857	3.796926
H	3.697483	-1.544473	5.125294
H	3.758134	6.429388	-4.799646
H	5.549815	6.371087	-3.537729
H	6.327881	-1.119336	-0.489935
H	6.607663	0.027461	-2.341010
H	-5.135262	6.638120	2.629844
H	-6.316020	7.132331	0.847620
H	-2.557510	5.875361	-5.993168
H	-2.156454	7.739081	-4.917783
H	-0.651067	-0.873241	-6.497039
H	0.651067	0.873241	-6.497039
H	-3.697483	1.544473	5.125294
H	-2.093484	2.241857	3.796926
H	-6.607663	-0.027461	-2.341010
H	-6.327881	1.119336	-0.489935
H	6.316020	-7.132331	0.847620
H	5.135262	-6.638120	2.629844

H	-3.758134	-6.429388	-4.799646
H	-5.549815	-6.371087	-3.537729
H	-5.726324	-5.829665	3.372561
H	-6.786102	-4.294038	2.232897
H	0.678402	-7.303152	5.702292
H	1.479694	-5.527530	6.675183
H	2.156454	-7.739081	-4.917783
H	2.557510	-5.875361	-5.993168
H	0.503774	-10.483963	0.549855
H	-1.491261	-10.116769	-0.290314

2

Ir	2.873193	0.070792	0.090731
Ir	-2.826491	0.006978	0.030224
H	2.204945	0.681479	1.466877
H	2.578725	1.432681	-0.844497
H	-2.516569	-1.338346	-0.910088
Ag	5.729188	-0.153393	0.363601
Ag	3.971964	-0.230177	2.715799
Ag	4.024273	-2.477613	0.774518
Ag	1.454201	-1.631201	1.922500
Ag	3.931785	-4.406161	-1.522783
Ag	4.189846	2.344832	1.097652
Ag	4.198304	-1.378574	-2.018398
Ag	4.538372	1.485745	-1.810995
Ag	1.609572	-2.267367	-1.018718
Ag	1.733787	0.291738	-2.524219
Ag	-0.011603	-0.034665	-0.076921
Ag	6.736919	1.556052	2.635980
Ag	1.235814	2.586334	0.184614
Ag	6.938674	2.600814	-0.441168
Ag	-1.298991	-2.724824	-0.024276
Ag	-1.506898	2.272308	-1.099261
Ag	-1.433251	1.736842	1.871970
Ag	-1.576884	-1.089116	2.326442
Ag	1.804018	-4.576025	1.007666
Ag	-4.114397	1.400456	-2.109936
Ag	-3.949447	2.593332	0.623902
Ag	-1.697708	-0.274257	-2.614841

Ag	-4.016352	0.442505	2.659564
Ag	-4.101245	-2.201943	1.192265
Ag	-4.459513	-1.493489	-1.801935
Ag	-5.666120	0.242848	0.266190
Ag	7.043919	-0.242151	-2.323947
Ag	-1.606584	4.638767	0.956325
Ag	-3.752805	4.446957	-1.728010
Ag	-6.823964	-2.565504	-0.404560
Ag	-6.768708	-1.369925	2.566370
Ag	-6.924344	0.217551	-2.464373
S	7.986644	-0.607000	1.783701
S	4.770985	-1.227446	4.924285
S	5.801522	-4.485519	0.346685
S	-0.041060	-2.401157	3.968584
S	4.503704	-3.303145	-3.812443
S	2.098200	-6.204282	-1.102757
S	4.545547	4.842166	1.518024
S	5.754753	3.759472	-2.508994
S	-0.134144	-3.715484	-2.360691
S	1.364961	0.784237	-4.948487
S	7.909110	3.762338	1.697229
S	5.282946	2.220002	4.651934
S	9.033999	1.104975	-1.169779
S	0.944826	4.742371	1.575741
S	3.486826	-4.870849	2.950358
S	-0.752961	-4.861894	1.498903
S	0.311188	3.650602	-2.390290
S	-0.363292	2.598114	4.050578
S	7.650426	-2.766927	-2.310996
S	6.219160	0.922358	-4.508120
S	-1.654653	-1.035539	-5.013361
S	-4.361642	3.268690	-3.970512
S	-5.680533	4.620148	0.079025
S	-5.085868	1.638060	4.659380
S	-4.450192	-4.695141	1.725918
S	-5.638549	-3.809287	-2.439886
S	-7.976073	0.748218	1.556915
S	-1.884332	6.180200	-1.200224

S	-3.444420	4.925277	2.751663
S	-7.521051	2.741172	-2.482165
S	-5.589660	-1.827713	4.798695
S	-7.831882	-3.682485	1.739374
S	-8.913966	-1.146581	-1.314538
S	-6.042300	-1.056553	-4.565608
P	9.430792	0.068997	0.531552
P	5.238384	0.530658	5.772161
P	5.029360	-5.588272	1.878502
P	-0.832792	-4.192731	3.416488
P	6.549755	-3.395505	-3.856031
P	0.460509	-5.642161	-2.174362
P	6.394896	5.004785	2.279538
P	6.630144	2.847136	-4.110082
P	0.294604	-0.843497	-5.487558
P	1.026677	3.902525	3.450104
P	-0.221933	5.587370	-2.220969
P	-6.404657	3.381566	-4.014126
P	-4.903129	5.715262	1.613277
P	-5.650101	-0.001117	5.671222
P	-6.303590	-4.808654	2.489191
P	-6.496342	-2.951508	-4.078615
P	-9.374397	0.013285	0.285863
H	10.217424	-1.055875	0.173376
H	10.363803	0.836873	1.276210
H	4.362137	0.813960	6.855478
H	6.466630	0.392903	6.476799
H	4.755205	-6.900466	1.404499
H	6.103214	-5.849317	2.769503
H	-2.192104	-4.234494	3.821248
H	-0.235666	-5.188888	4.230416
H	6.917514	-2.749152	-5.065956
H	6.905288	-4.739437	-4.146139
H	-0.624388	-6.417205	-1.688262
H	0.609233	-6.165905	-3.484924
H	6.367909	4.965217	3.699522
H	6.845210	6.332146	2.052524
H	6.289837	3.619177	-5.251920

H	8.031642	3.074637	-4.043571
H	0.384480	-0.936973	-6.902177
H	0.998527	-2.017599	-5.102829
H	1.073290	4.994935	4.356184
H	2.344209	3.381908	3.579175
H	-0.316382	6.130230	-3.529176
H	0.857814	6.339834	-1.688441
H	-6.775558	2.754378	-5.233229
H	-6.755832	4.730591	-4.285572
H	-5.990756	6.059330	2.458637
H	-4.535962	6.999333	1.124424
H	-6.945113	0.221836	6.217395
H	-4.897637	-0.125837	6.871693
H	-6.722541	-6.163811	2.418631
H	-6.292190	-4.598681	3.894639
H	-7.903158	-3.139452	-3.999119
H	-6.180459	-3.783669	-5.184904
H	-10.356228	-0.683681	1.037781
H	-10.122266	1.121870	-0.188136

3

Ir	-2.856533	0.006426	-0.029328
Ir	2.853322	0.009889	-0.030079
H	-2.545555	1.344525	0.924456
H	2.528865	-1.342611	0.895519
Ag	-5.703108	-0.172502	-0.314203
Ag	-3.988946	-0.362193	-2.682302
Ag	-4.024618	-2.531571	-0.644827
Ag	-1.526201	1.151981	-2.260351
Ag	-1.478922	-1.729067	-1.857578
Ag	-3.891295	-4.394989	1.683984
Ag	-4.079603	2.255057	-1.175277
Ag	-4.176693	-1.356822	2.101474
Ag	-4.497719	1.524074	1.783605
Ag	-1.571117	-2.280084	1.100711
Ag	-1.693516	0.261709	2.581273
Ag	0.003588	0.002219	0.087530
Ag	-6.742056	1.463417	-2.607964
Ag	-1.265656	2.723658	0.156609

Ag	-6.854272	2.621446	0.380696
Ag	1.284687	-2.709842	0.051807
Ag	1.573351	2.241322	1.199481
Ag	1.475167	1.811758	-1.790272
Ag	1.525400	-1.052200	-2.307072
Ag	-1.777090	-4.620257	-0.875041
Ag	4.189322	1.296940	2.144132
Ag	4.019031	2.571198	-0.550286
Ag	1.720333	-0.340205	2.611746
Ag	3.985653	0.476895	-2.665946
Ag	4.077268	-2.196151	-1.256862
Ag	4.492467	-1.577872	1.721286
Ag	5.700013	0.198350	-0.309714
Ag	-6.989785	-0.176719	2.387278
Ag	1.762387	4.653996	-0.702283
Ag	3.877631	4.348215	1.848252
Ag	6.848730	-2.623816	0.281549
Ag	6.740772	-1.356914	-2.660705
Ag	6.998892	0.094273	2.386926
S	-7.996860	-0.657140	-1.661058
S	-5.015317	-1.525353	-4.727525
S	-5.767129	-4.539419	-0.187709
S	-0.049754	2.649504	-3.810225
S	0.043467	-2.502199	-3.902241
S	-4.488770	-3.237050	3.941091
S	-2.062246	-6.204341	1.270339
S	-4.444727	4.761512	-1.617250
S	-5.672168	3.815637	2.449362
S	0.196293	-3.703466	2.449762
S	-1.305619	0.749651	5.003797
S	-7.823117	3.763237	-1.772305
S	-5.506679	1.943334	-4.803316
S	-8.966336	1.198116	1.227253
S	-0.802541	4.959194	-1.221768
S	-0.203895	3.616026	2.581053
S	-3.467076	-4.995390	-2.798088
S	0.787958	-4.897603	-1.389419
S	-7.627850	-2.697704	2.421321

S	-6.177274	1.053430	4.544865
S	1.731672	-1.045917	5.024616
S	4.522805	3.106815	4.045920
S	5.747970	4.570105	-0.028820
S	5.010979	1.708785	-4.670276
S	4.439458	-4.686851	-1.793428
S	5.666885	-3.895267	2.302456
S	7.994591	0.725476	-1.635161
S	2.040575	6.162468	1.492355
S	3.443086	5.111129	-2.618256
S	7.637318	2.611674	2.457354
S	5.501402	-1.755519	-4.869921
S	7.817878	-3.688487	-1.912077
S	8.969005	-1.244089	1.175388
S	6.149304	-1.198890	4.488834
P	-9.409612	0.081911	-0.409292
P	-5.567720	0.134796	-5.711852
P	-4.997264	-5.683530	-1.691094
P	-0.872796	4.396084	-3.173810
P	0.868584	-4.266824	-3.321046
P	-6.535795	-3.315236	3.976527
P	-0.399767	-5.631797	2.296536
P	-6.266403	4.892804	-2.452779
P	-6.585619	2.957198	4.060534
P	-0.213619	-0.868777	5.525261
P	0.374661	5.552234	2.492143
P	6.567802	3.205359	4.039397
P	4.969102	5.766612	-1.486164
P	5.562031	0.084379	-5.713206
P	6.259821	-4.788454	-2.635870
P	6.555188	-3.090835	3.954146
P	9.408969	-0.057277	-0.411441
H	-10.191801	-1.019941	0.023106
H	-10.358348	0.815426	-1.168871
H	-4.805950	0.279751	-6.904116
H	-6.859461	-0.073548	-6.271177
H	-4.713264	-6.978247	-1.176282
H	-6.076304	-5.978878	-2.564960

H	-0.285325	5.440670	-3.931896
H	-2.233967	4.444819	-3.573143
H	2.232939	-4.298391	-3.711355
H	0.289124	-5.287087	-4.117434
H	-6.902001	-2.651714	5.177679
H	-6.902232	-4.652861	4.282126
H	0.672951	-6.412398	1.792204
H	-0.515817	-6.142213	3.615722
H	-6.201833	4.702287	-3.859579
H	-6.679548	6.249340	-2.378374
H	-6.283913	3.778367	5.178989
H	-7.985647	3.173726	3.943397
H	-0.280078	-0.962648	6.941026
H	-0.910881	-2.049214	5.149001
H	0.485459	6.016936	3.828344
H	-0.696825	6.348704	2.009753
H	6.965618	2.533821	5.225955
H	6.932266	4.542480	4.349528
H	6.045347	6.103793	-2.348355
H	4.676275	7.038294	-0.921214
H	6.853332	0.312206	-6.265898
H	4.799271	-0.018013	-6.909271
H	6.671034	-6.147496	-2.615238
H	6.193850	-4.542831	-4.034077
H	7.956238	-3.310748	3.857367
H	6.228191	-3.942174	5.042406
H	10.363327	-0.752397	-1.199542
H	10.183416	1.030410	0.068152
